# The FMRpolyGlycine Protein Mediates Aggregate Formation and Toxicity Independent of the CGG mRNA Hairpin in a Cellular Model for FXTAS

**DOI:** 10.3389/fgene.2019.00249

**Published:** 2019-03-28

**Authors:** Gry Hoem, Kenneth Bowitz Larsen, Aud Øvervatn, Andreas Brech, Trond Lamark, Eva Sjøttem, Terje Johansen

**Affiliations:** ^1^Molecular Cancer Research Group, Department of Medical Biology, University of Tromsø – The Arctic University of Norway, Tromsø, Norway; ^2^Department of Molecular Cell Biology, Institute for Cancer Research, Oslo University Hospital, Oslo, Norway; ^3^Faculty of Medicine, Centre for Cancer Cell Reprogramming, Institute of Clinical Medicine, University of Oslo, Oslo, Norway

**Keywords:** FXTAS, CGG repeat expansion, RNA hairpin, RAN translation, polyglycine, protein degradation

## Abstract

Fragile X-associated tremor/ataxia syndrome (FXTAS) is a neurodegenerative disorder caused by a CGG-repeat expansion in the 5′ UTR of the FMR1 gene on the X-chromosome. Both elevated levels of the expanded FMR1 mRNA and aberrant expression of a polyglycine protein (FMRpolyG) from the CGG-repeat region are hypothesized to trigger the pathogenesis of FXTAS. While increased expression of FMRpolyG leads to higher toxicity in FXTAS models, the pathogenic effect of this protein has only been studied in the presence of CGG-containing mRNA. Here we present a model that allows measurement of the effect of FMRpolyG-expression without co-expression of the corresponding CGG mRNA hairpin. This allows direct comparison of the effect of the FMRpolyG protein *per se*, vs. that of the FMRpolyG protein together with the CGG mRNA hairpin. Our results show that expression of the FMRpolyG, in the absence of any CGG mRNA, is sufficient to cause reduced cell viability, lamin ring disruption and aggregate formation. Furthermore, we found FMRpolyG to be a long-lived protein degraded primarily by the ubiquitin-proteasome-system. Together, our data indicate that accumulation of FMRpolyG protein *per se* may play a major role in the development of FXTAS.

## Introduction

Fragile X-associated tremor/ataxia syndrome (FXTAS, #300623) is a neurodegenerative disorder affecting individuals with a premutation CGG-expansion (55–200 repeats) in the 5′ untranslated region (UTR) on the Fragile X mental retardation 1 (FMR1) gene on the X-chromosome (Hagerman et al., 2001). The main clinical features of FXTAS include motor symptoms such as gait ataxia and intention tremor, often accompanied by Parkinsonism and cognitive impairment (Hagerman et al., 2001; Jacquemont et al., 2003). The prevalence of the FMR1 premutation causing FXTAS is 1 in ~290 women and 1 in ~850 men (Hunter et al., 2014), but the penetrance is around 40% in men and 8–16% in women aged over fifty (Jacquemont et al., 2004; Coffey et al., 2008; Rodriguez-Revenga et al., 2009).

Full-mutation expansions of the FMR1 CGG repeat region (>200 CGGs) usually result in silencing of the gene and lack of FMR1 mRNA and protein (FMRP). This absence, or very reduced level of FMRP, is the cause of the neurodevelopmental disorder Fragile X Syndrome (#300624). Contrary to what is seen upon the full-mutation, premutation carriers have 2–8-fold increased levels of FMR1 mRNA (Tassone et al., 2000) and normal or slightly reduced levels of FMRP (Kenneson et al., 2001). This increased FMR1-mRNA level, coupled with studies revealing the presence of the expanded FMR1-mRNA inside intranuclear inclusions in patient materials (Greco et al., 2002; Tassone et al., 2004; Iwahashi et al., 2006), led to the development of an RNA-gain-of-function hypothesis for the FXTAS pathogenesis (reviewed in Hagerman and Hagerman, 2004). Several studies have since demonstrated that expression of CGG repeats in the premutation range leads to features of toxicity and inclusion formation in both cellular and animal model systems (Jin et al., 2003; Willemsen et al., 2003; Arocena et al., 2005; Entezam et al., 2007; Hashem et al., 2009; Hoem et al., 2011; Hukema et al., 2014). The CGG mRNA forms a strong hairpin structure (Handa et al., 2003; Zumwalt et al., 2007), but the exact mechanisms by which the expanded CGG mRNA hairpin induces pathogenic events, have not been fully elucidated. Among the proposed pathways are sequestration of specific proteins by the expanded CGG mRNA hairpin itself (Jin et al., 2007; Sofola et al., 2007; Sellier et al., 2010, 2013), R-loop formation (Loomis et al., 2014), and excessive induction of DNA-damage response (Hoem et al., 2011). A more recently proposed mechanism for the FXTAS-pathogenesis, is repeat-associated non-AUG (RAN) translation. RAN translation across microsatellite repeat expansions was first described by Zu et al. (2011). In 2013 Todd et al. found that RAN translation also occurs across the expanded CGG repeat FMR1-mRNA, causing formation of FMRpolyAlanine and FMRpolyGlycine (FMRpolyG) proteins (Todd et al., 2013). While conventional translation of the FMR1 mRNA initiates at an AUG codon downstream of the CGG repeats, RAN translation starts primarily at a near-cognate start codon (ACG) upstream of the repeats, thus including the repeat stretch in the translation product (Todd et al., 2013; Kearse et al., 2016; Sellier et al., 2017). The resulting aberrant expression of the FMRpolyG protein from the CGG-expansion appears to have a toxic effect (Todd et al., 2013). Furthermore, the FMRpolyG protein colocalizes with the ubiquitin positive intranuclear inclusions found in FXTAS patients (Todd et al., 2013; Buijsen et al., 2014; Sellier et al., 2017). Interestingly, Sellier et al. (2017) recently described a mouse model where they compared expression of expanded CGG repeats without formation of the RAN-translation product FMRpolyG, to expression of both the expanded CGG repeats and the FMRpolyG protein. They clearly demonstrated that expression of CGG mRNA without formation of FMRpolyG, resulted in a milder phenotype than that observed when both components were expressed. In addition, they showed that expression of the C-terminus of the FMRpolyG protein alone had a toxic effect in cell culture. However, the consequences of expressing the entire FMRpolyG protein itself, without co-expression of the hairpin-forming CGG mRNA, have not been investigated.

In this study we establish a system allowing expression of (a) both the FMRpolyG protein and the CGG mRNA hairpin and (b) the FMRpolyG protein without any CGG mRNA. We show that expressing the FMRpolyG protein outside the context of the CGG repeats, and thus without any CGG RNA hairpin, leads to aggregate formation and cellular toxicity. Furthermore, FRAP analyses demonstrated that FMRpolyG is completely immobilized in the aggregates. Finally, we provide evidence for the FMRpolyG being a long-lived protein degraded through the ubiquitin-proteasomal system (UPS). This is in line with previous observations of reduced neurodegeneration upon induction of the UPS (Oh et al., 2015) and points at the UPS as a potential target for therapeutic intervention in FXTAS.

## Results

### Generation of a Construct Expressing FMRpolyG-GFP Without CGG RNA Hairpin Formation

**Figure 1:**
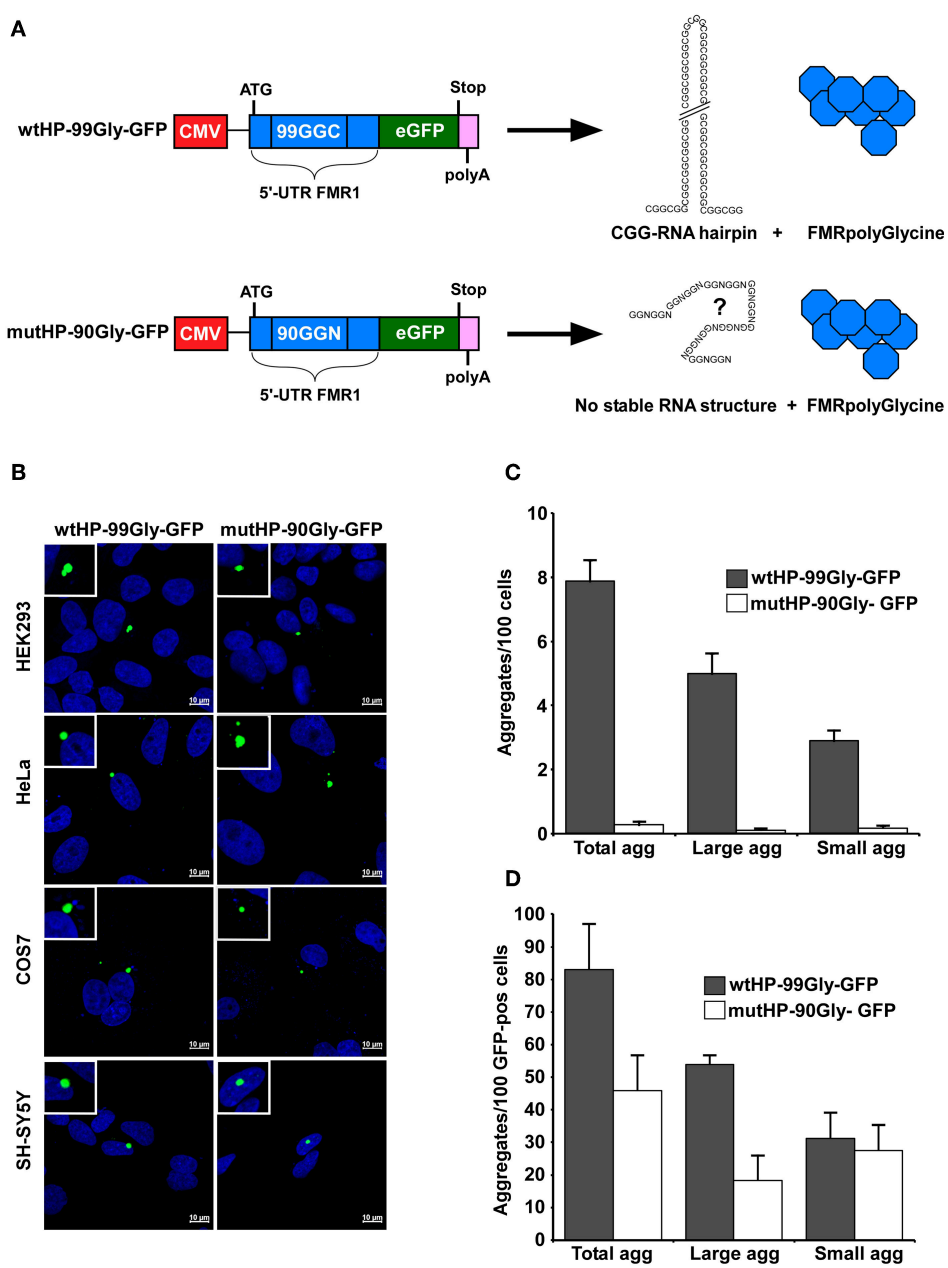
The FMRpolyG protein itself causes aggregate formation in several cell lines. **(A)** Schematic representation of the wtHP-99Gly-GFP and mutHP-90Gly-GFP constructs used in this study. Both contain the part of the FMR1 5′UTR that encodes the entire FMRpolyG protein (shown in blue). The wtHP-99Gly-GFP construct gives rise to both a CGG RNA hairpin and the FMRpolyGlycine protein. In mutHP-90Gly-GFP, the CGGs have been substituted with alternative glycine codons to abolish hairpin formation. **(B)** Confocal images of GFP-positive aggregates in the indicated cell lines, transfected with wtHP-99Gly-GFP or mutHP-90Gly-GFP and analyzed 24–48 h after transfection. **(C,D)** Quantification of FMRpolyG-GFP aggregates in HEK293 cells 24 h after transfection, presented as number of aggregates per 100 cells **(C)** or 100 GFP-positive cells **(D)**. Untransfected cells and cells transfected with GFP-C1 were used as negative controls. Small aggregates range from 1 to 3 μm^2^ in size, while large aggregates are those over 3 μm^2^. Calculations are based on three individual experiments, each including >4,500 cells for each plasmid. Error bars represent standard deviation (SD).

While several model systems for FXTAS have shown that FMRpolyG expression increases toxicity, this has only been studied in the presence of CGG repeat DNA and RNA (Todd et al., 2013; Oh et al., 2015; Sellier et al., 2017). A toxic effect of the entire protein alone has not been demonstrated. To address this, we constructed a plasmid where the CGG repeats were replaced by a sequence containing 90 alternative glycine codons in the +1 frame. Note that the CGGs are read “GGC” in the +1 reading frame coding for the FMRpolyG protein. We refer to this region as the CGG repeats, to point out that we are describing the area of the FMR1-gene called the “CGG repeat region” in the FXTAS literature. The complete sequence encoding the FMRpolyG protein without CGG repeats, was inserted into a plasmid downstream of a CMV-promoter and in frame with a GFP-tag at the C-terminal end. The resulting plasmid was named mutatedHairPin-90Glycine-GFP (mutHP-90Gly-GFP). This was used in parallel with a plasmid expressing the native RNA hairpin-forming CGG repeat sequence (Sellier et al., 2017), here called wildtypeHairPin-99Glycine-GFP (wtHP-99Gly-GFP) (Figure 1A). ATG start codons were inserted to ensure expression from both constructs. With the exception of intended substitution of the CGG repeats, the FMRpolyG encoding sequences are identical in both plasmids (Supplementary Figure 1A). The wtHP-99Gly-GFP construct has already been demonstrated to cause features of FXTAS in cell culture (Sellier et al., 2017).

To determine the potential of the mutHP-90Gly to form an RNA hairpin structure, the polyG-sequences of both mutHP-90Gly and wtHP-99Gly were analyzed using the bioinformatic tools RNAfold (http://rna.tbi.univie.ac.at/cgi-bin/RNAWebSuite/RNAfold.cgi) and RNAstructure (https://rna.urmc.rochester.edu/RNAstructureWeb/Servers/Predict1/Predict1.html). The wtHP-99Gly has a strong potential to form a hairpin structure, while this is not the case for mutHP-90Gly (Supplementary Figure 2). Not only is the predicted secondary structure vastly different between the two, but the free energy and the frequency of the minimal free energy (MFE) structures varies by >40-fold between them, with the CGG RNA hairpin from the wtHP-99Gly-construct standing out as the only stable secondary structure. While such predictions provide some information as to what the expected structure could be, it is important to keep in mind that they are not sufficient to completely exclude secondary structures formed by the mRNA. It is beyond the scope of this paper to determine the exact secondary structure of these mRNAs. However, since all the bioinformatic prediction tools show that the CGG-mRNA forms a stable hairpin, while the GGN-mRNA does not, we concluded that the latter forms a different and less stable secondary structure. Since the mutHP-90Gly sequence does not contain the CGGs, it cannot form a CGG-hairpin.

Sellier et al. (2017) have demonstrated that expression of the FMRpolyG protein together with the CGG RNA hairpin causes aggregate formation in both a mouse model system, neuronal cell lines and HEK293 cells. We first confirmed the results by transiently transfecting HEK293 cells with the same plasmid as used by Sellier et al., namely wtHP-99Gly-GFP. Next, we tested whether the mutHP-90Gly-GFP–plasmid was able to induce aggregate formation in cells. Importantly, aggregate formation was detected not only in cells transfected with wtHP-99Gly-GFP, but also in those transfected with mutHP-90Gly-GFP (Figure 1B). In addition, both constructs induced aggregate formation in different cell lines, including HeLa, COS7, and neuronal SH-SY5Y cells (Figure 1B). This demonstrates that the FMRpolyG protein alone, without co-expression of the CGG RNA hairpin, can give rise to aggregates in various cell lines.

### The wtHP-99Gly-GFP Construct Gives Many Fold Higher Protein Expression Levels Than the mutHP-90-Gly-GFP Construct

Interestingly, we found both the size and number of aggregates, and number of cells expressing detectable levels of the GFP-tagged FMRpolyG protein, to be several folds higher in cells transfected with wtHP-99Gly-GFP, compared to those transfected with mutHP-90Gly-GFP. We quantified the number of aggregates both relative to the total number of cells, and to the number of GFP-positive cells visualized by confocal microscopy. To avoid overexposure of GFP-positive aggregates during confocal microscopy, settings resulted in a high threshold of GFP-intensity required to count a cell as GFP-positive. The threshold is the same for wtHP-99Gly-GFP and mutHP-90Gly-GFP expressing cells. The number of aggregates per 100 total cells is ~30-fold higher in wtHP-99Gly-GFP than in mutHP-90Gly-GFP expressing cells (Figure 1C). This ratio is reduced to just ~1.8-fold when comparing only the GFP-positive cells (Figure 1D). Hence, the elevated protein-expression levels of wtHP-99Gly-GFP compared to mutHP-90Gly-GFP may explain the increased aggregate formation in cells transfected with wtHP-99Gly-GFP. Importantly, aggregate formation was also seen sporadically upon expression of both constructs when the ATG is replaced with the native ACG-codon (Supplementary Figures 2B,C).

The mutHP-90Gly-GFP construct used in this study, contained an around 100 nt insertion 5′ to the polyGly ATG start-codon (Supplementary Figure 1A). To elucidate if these inserted nucleotides had any impact on the rate of transcription/translation, a new construct with a 5′ region identical to the wtHP-99Gly-GFP was established (Supplementary Figure 1B). We assessed the aggregate formation and FMRpolyG mRNA levels in the cells expressing this new construct (Supplementary Figure 3). Importantly, removal of the around 100 nucleotides insertion did not affect aggregate formation or the FMRpolyG mRNA levels, clearly indicating that this insertion had no impact on the expression from the mutHP-90Gly-GFP construct (Supplementary Figure 3).

**Figure 2:**
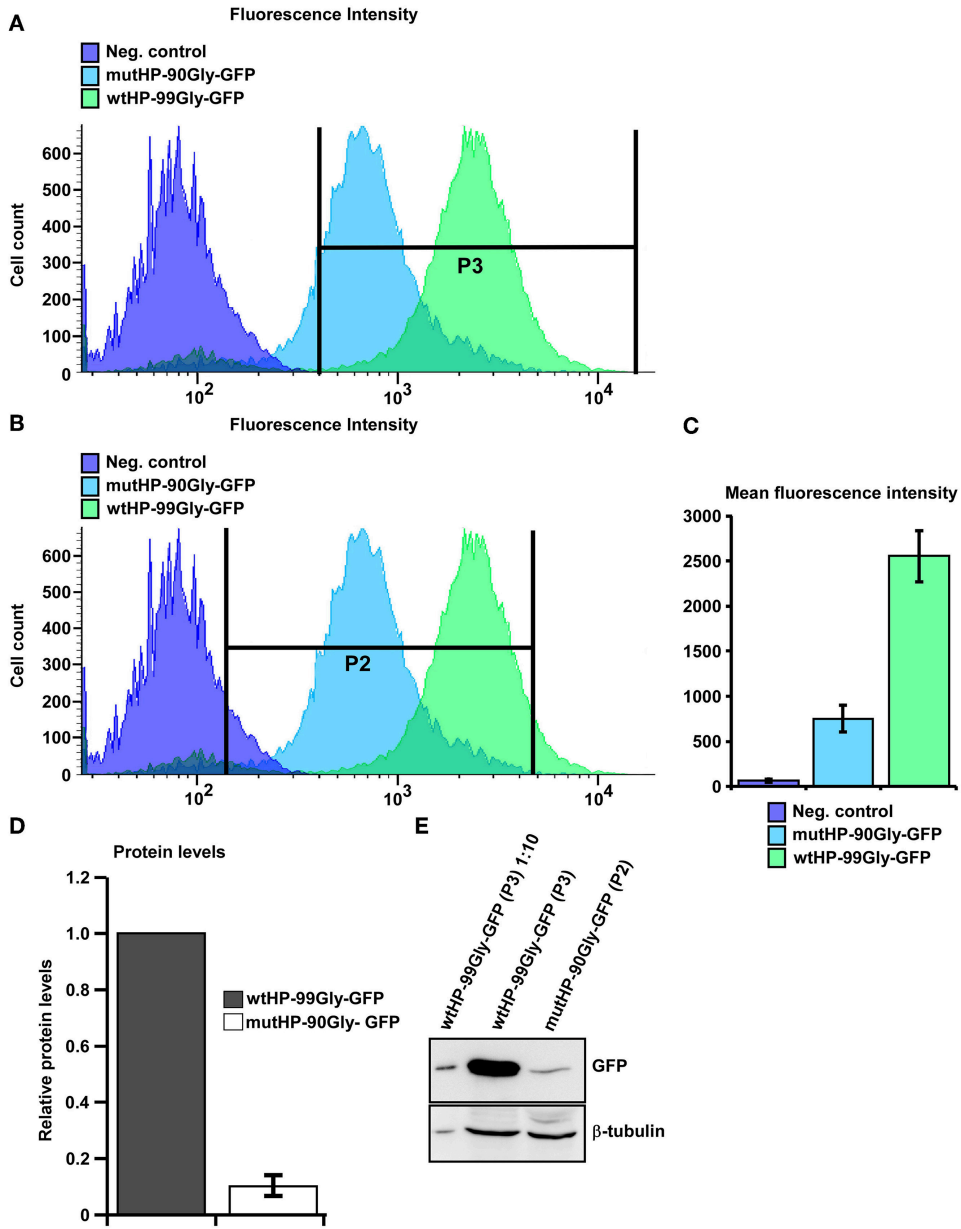
Expression of wtHP-99Gly-GFP results in several fold higher protein levels than that of mutHP-90-Gly-GFP expression. **(A,B)** Merge of flow curves showing the differences in GFP fluorescence intensity in HEK-FlpIn cells expressing no GFP-tagged protein (negative control, purple), mutHP-90Gly-GFP (blue) and wtHP-99Gly-GFP (green). Flow analysis and sorting was performed separately on each cell population 24 h after induction of expression. Note the logarithmic scale on the X-axis. GFP-positive cells expressing wtHP-99Gly-GFP were sorted into gate P3. GFP-positive cells expressing mutHP-90Gly-GFP were sorted into gate P2. **(C)** The graph demonstrates the large difference in mean fluorescence (GFP) intensity between mutHP-90Gly-GFP and wtHP-99Gly-GFP expressing cells. **(D)** The graph shows the difference in FMRpolyG-GFP protein levels in HEK-FlpIn cells stably expression mutHP-90Gly-GFP and wtHP-99Gly-GFP. The cells were sorted using flow with the gates illustrated in **(A,B)**. For mutHP-90Gly-GFP cells expressing GFP-levels above the threshold in gate P2 were included, while wtHP-99Gly-GFP expressing cells included are those above the threshold in gate P3. The graph is based on WB-quantification of sorted cells from the two above mentioned cell populations. **(E)** Western blot of sorted cells expressing wtHP-99Gly-GFP or mutHP-90Gly-GFP. Protein levels of wtHP-99Gly-GFP expressing cell is set to 1 in each experiment. Data in A-D is based on three independent experiments were cells were subject to flow, sorted and lysed 24 h after expression was induced.

Next, we established two Flp-In-T-REx 293 (HEK-FlpIn) cell lines with inducible expression of wtHP-99Gly-GFP or mutHP-90Gly-GFP, respectively. Induction of FMRpolyG expression in these cells demonstrated the same pattern with higher levels of GFP-positive cells and more cells with aggregates in the wtHP-99Gly-GFP cells compared to the mutHP-90Gly-GFP cells. This prompted us to monitor more closely the FMRpolyG-GFP protein levels in the two different cell populations. We first performed flow analyses on the two HEK-FlpIn cell lines, 24 h after expression was induced. For both cell lines, around 90% of the cells were GFP-positive (Figures 2A,B), indicating that there is no difference in the portion of cells expressing the GFP-tagged protein. However, the GFP fluorescence intensity of the wtHP-99Gly-GFP expressing cells was around 5-fold higher than in the GFP fluorescence intensity of the mutHP-99Gly-GFP cells (Figures 2A–C). To verify if the GFP fluorescence intensity pictured the expression level of the FMRpolyG protein, GFP-positive cells from both cell lines were sorted and harvested, and the FMRpolyG protein levels quantitated by Western blotting (Figures 2D,E). In line with the flow and imaging analyses, wtHP-99Gly-GFP-expressing cells demonstrated ~10-fold higher FMRpolyG protein levels compared to mutHP-GFP expressing cells (Figure 2D).

To investigate if the enhanced protein expression from the wtHP-99Gly-GFP expressing cells is due to enhanced wtHP-99Gly-GFP mRNA levels, FMRpolyG-GFP-protein and mRNA levels were quantitated both in transiently transfected and in the induced HEK-FlpIn cells (Supplementary Figure 4). qPCR analyses performed on RNA from cells harvested 24 h post transfection with either construct, revealed ~ 22 times more GFP-mRNA in cells transfected with wtHP-99Gly-GFP, compared to those transfected with mutHP-90Gly-GFP (Supplementary Figure 4A). Furthermore, the FMRpolyG protein levels measured by Western blot was ~5-fold higher in wtHP-99Gly-GFP cells than in mutHP-90-Gly-GFP cells (Supplementary Figures 4B,C). For the induced HEK-FlpIn cell lines, levels of GFP-mRNA and FMRpolyG protein (measured by Western blot) were, respectively, ~11 and 13-folds higher for the cell line expressing wtHP-99GGC-GFP than the cell line expressing mutHP-90Gly-GFP (Supplementary Figures 4D–F). In total, these results show that the wtHP-99Gly-GFP expression construct leads to several fold higher mRNA and protein levels compared to the mutHP-90Gly-GFP expression construct, even when the same number of cells are expressing each construct. This could point toward the RNA hairpin being important for high levels of FMRpolyG mRNA, leading to high level of protein expression and aggregate formation. Importantly, the capacity for aggregate formation apparently directly correlates with the expression level of the FMRpolyG protein, though there is no evidence that this correlation is linear.

### Transient Transfection and Stable Expression of wtHP-99Gly-GFP and mutHP-90Gly-GFP Result in Similar Kinetics of FMRpolyG-GFP Aggregate Formation

Despite differences in mRNA levels and protein levels during both transient transfections and stable expression, both wtHP-99Gly-GFP and mutHP-90Gly-GFP expression led to aggregate formation in all cell lines tested.

**Figure 3:**
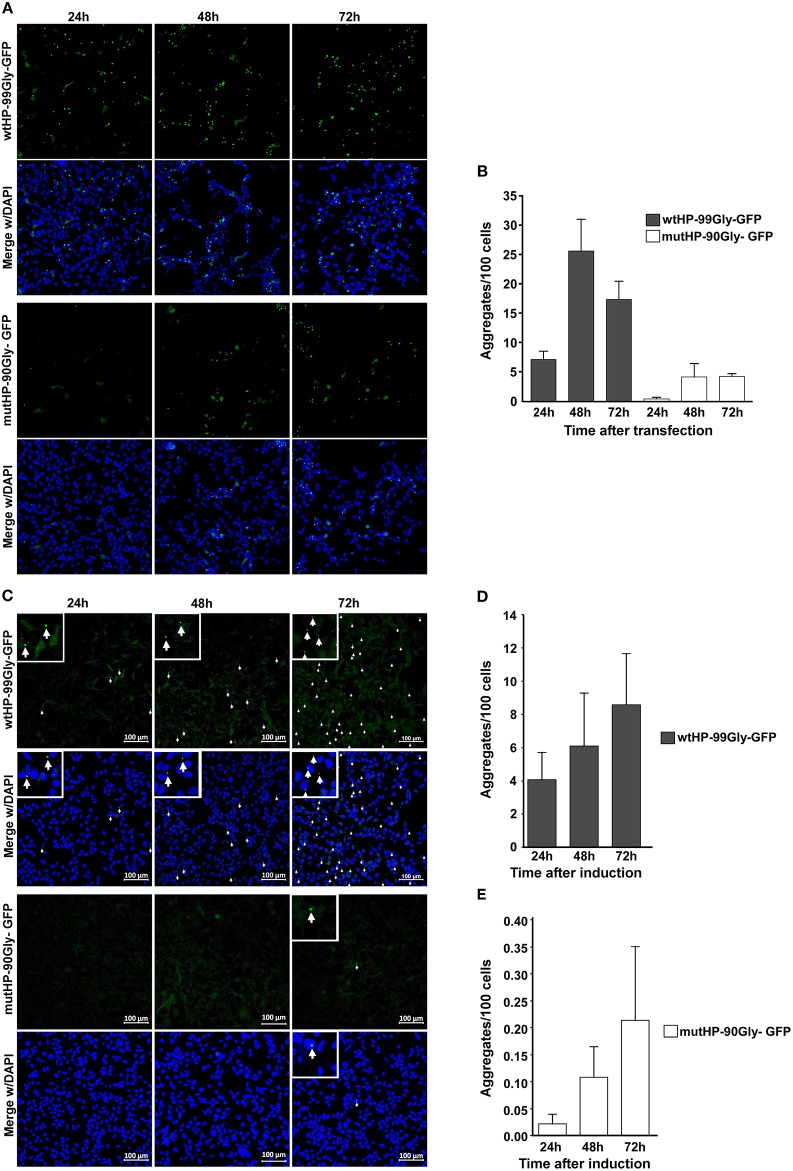
Both transient transfection and stable expression of wtHP-99Gly-GFP and mutHP-90Gly-GFP results in a similar kinetics of FMRpolyG-GFP aggregate formation. **(A)** Representative confocal images of transfected HEK293 cells, displaying aggregate formation 24, 48, and 72 h after transfection with either wtHP-99Gly-GFP (upper panel) or mutHP-90Gly-GFP (lower panel). Untransfected cells and cells transfected with GFP-C1 were used as negative controls. **(B)** Quantification of FMRpolyG-GFP aggregates formed 24, 48, and 72 h after transfection. **(C)** Confocal images of FMRpolyG-GFP aggregate formation in HEK-FlpIn cells stably expressing wtHP-99Gly-GFP (upper panel) or mutHP-90Gly-GFP (lower panel) from a tetracycline-inducible promoter. Doxycycline was added 24, 48, or 72 h prior to fixation. **(D,E)** Quantification of FMRpolyG-GFP aggregates formed in experiments shown in **(B)**. Error bars in **(B,D,E)** represent SD of averages from a minimum of three independent experiments.

In order to determine whether the wt and mutated construct has similar capacity to form aggregate over time, cells were fixed at 24, 48, and 72 h after transfection. Although wtHP-99Gly-GFP resulted in the highest number of aggregates and GFP-positive cells at every time point, the two constructs displayed similar aggregation kinetics (Figures 3A,B). Notably, aggregates were detected already 7 h after transfection for the wtHP-99Gly-GFP construct, and after 15 h for cells transfected with mutHP-90Gly-GFP (Supplementary Videos 1, 2).

Next, aggregate formation was analyzed by time-course experiments in the inducible HEK-FlpIn cell lines. Cells were fixed and imaged 24-, 48-, and 72 h after induction. Similar to the transient transfections, the highest number of aggregates was observed in wtHP-99Gly-GFP-expressing cells, at all time-points (Figures 3C–E). However, the pattern of increasing aggregate formation over time is the same for the two cell lines. In conclusion, both during transient transfection and upon stable expression, the wtHP-99Gly-GFP construct results in more mRNA, more FMRpolyG-GFP protein and a stronger capacity for aggregate formation, at all time points. This supports the notion that the CGG hairpin increases expression or stability of its own mRNA, and thus the amount of FMRpolyG expressed in cells. However, here we demonstrate that also a low, stable expression of the FMRpolyG protein, without a co-expressed CGG mRNA hairpin, has the ability to form aggregates in cells. Interestingly, despite the difference in absolute numbers of aggregates, the pattern of increasing aggregate formation over time is very similar whether cells express wtHP-99Gly-GFP or mutHP-90Gly-GFP.

### The FMRpolyG Protein Expression Induces Formation of Intranuclear Aggregates

**Figure 4:**
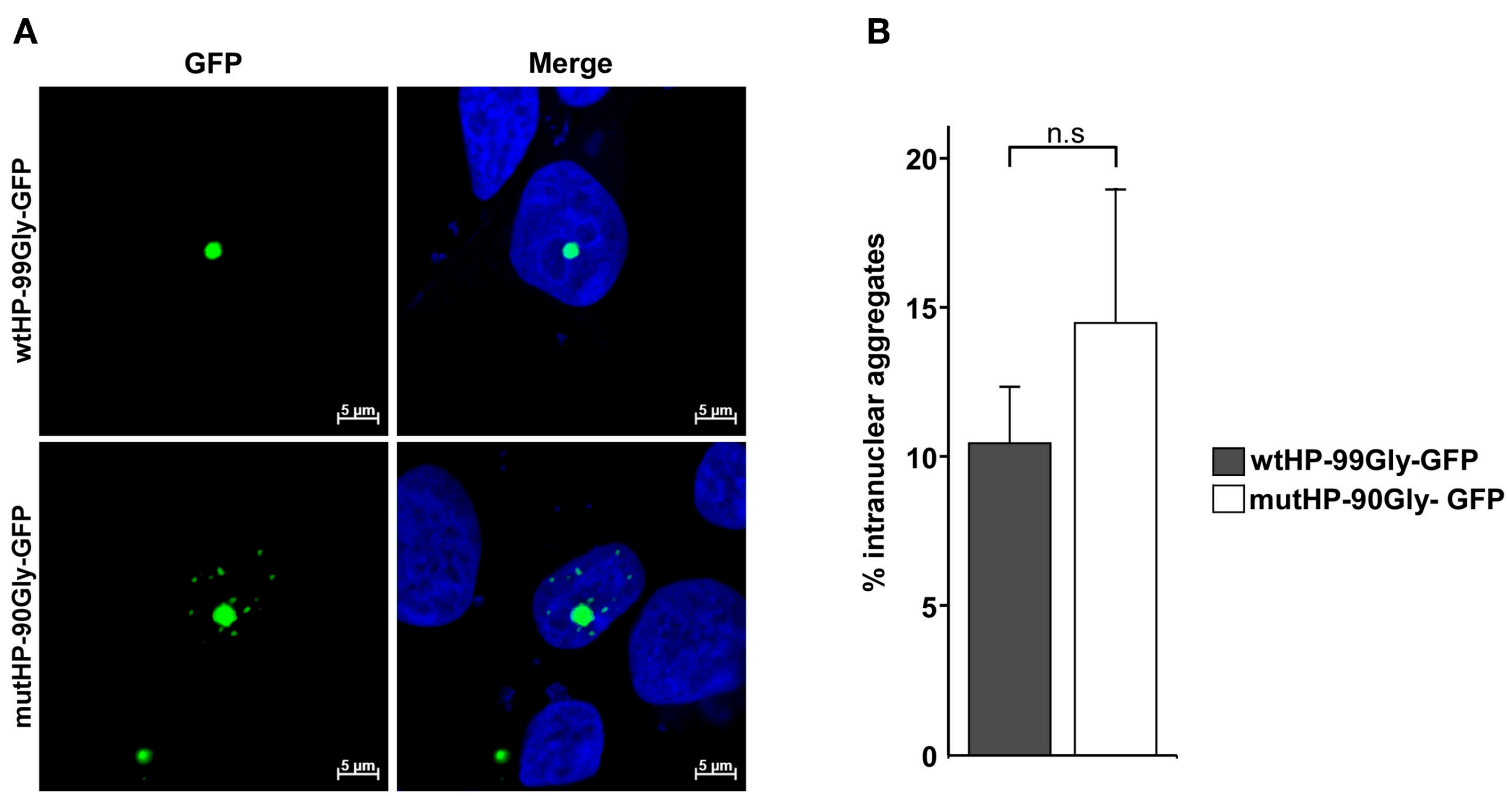
The FMRpolyG protein expression induces formation of intranuclear aggregates. **(A)** Representative confocal images of intranuclear aggregates in HEK293 cells transfected with wtHP-99Gly-GFP (upper panel) or mutHP-90Gly-GFP (lower panel). Untransfected cells and cells transfected with GFP-C1 were used as negative controls. **(B)** Quantification of aggregates that are intranuclear 24 h post transfection. The graphs represent the percentage of aggregates that are intranuclear, based on three individual experiments, each including a minimum of 45 aggregates per construct.

The neuropathological hallmark of FXTAS is the presence of intranuclear aggregates in both the CNS and peripheral tissues (Greco et al., 2002; Hunsaker et al., 2011). Formation of intranuclear aggregates by expression from the wtHP-99Gly-containing construct, has already been demonstrated (Sellier et al., 2017). In order to determine whether this was also the case for mutHP-90Gly-GFP, we used confocal microscopy to identify aggregates in cells transfected with either wtHP-99Gly-GFP or mutHP-90Gly-GFP. Importantly, the mutHP-90Gly-GFP construct lead to formation of the same portion of intranuclear aggregates as the wtHP-99Gly-GFP construct. We observed that around 10–15% of aggregates were intranuclear 24 h after transfection (Figures 4A,B). This clearly demonstrates that mutHP-90Gly has a similar potential as wtHP-99Gly to form intranuclear aggregates. Hence, this feature seems to be independent of the CGG mRNA hairpin.

### The FMRpolyG Protein Reduces Cell Viability and Disrupts the Lamin Architecture

**Figure 5:**
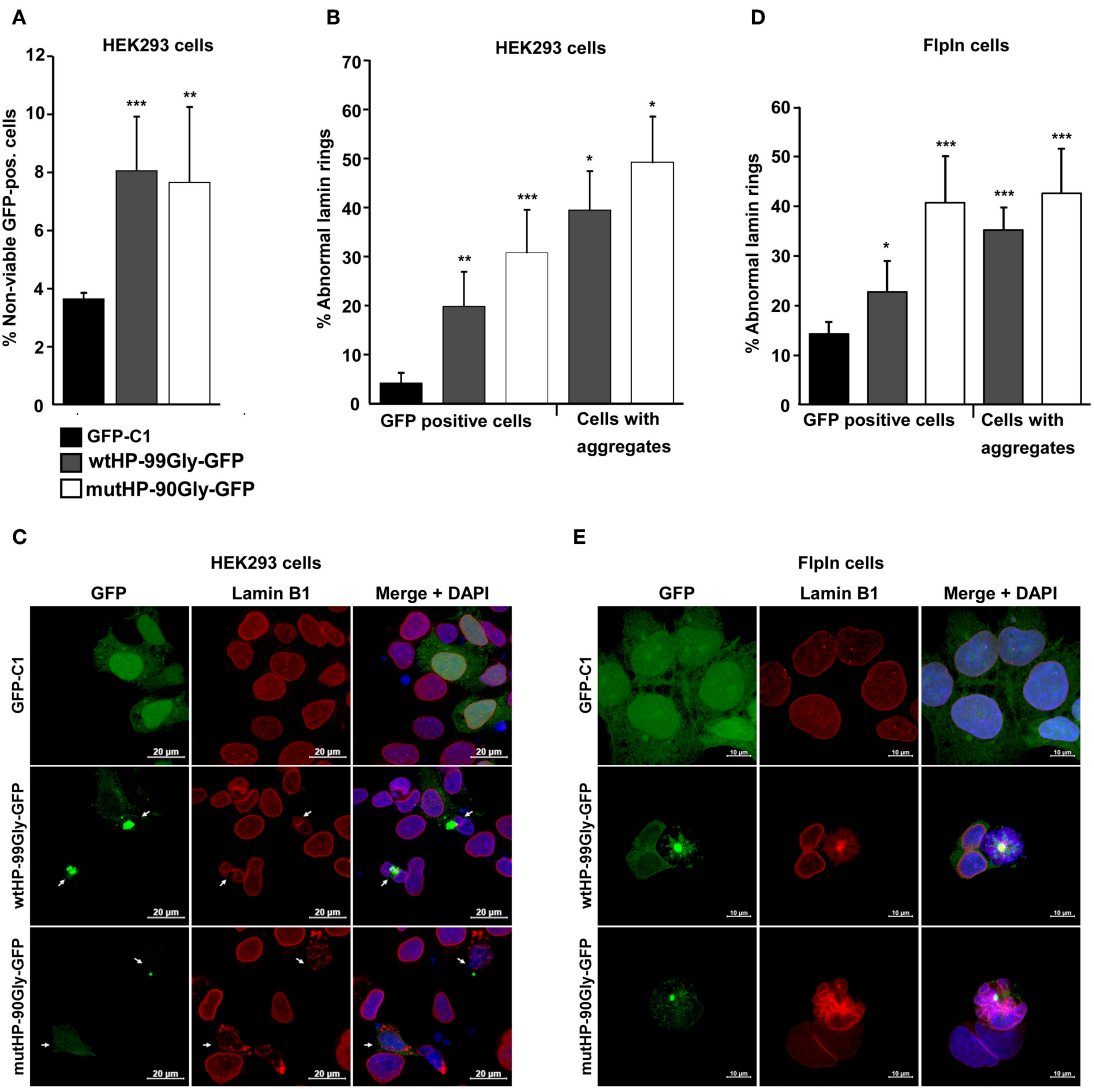
The FMRpolyG protein reduces cell viability and disrupts the lamin architecture. **(A)** Effect of FMRpolyG on cell viability. HEK293 cells were transfected with the indicated constructs, and cell death measured in GFP positive cells. Cell transfected with GFP-C1 were used as negative controls. Cells were counted as non-viable based on the incorporation of propidium iodide detected by FACS. For each transfection >50 000 cells were counted per experiment. ^***^p < 0.001; ^**^p < 0.01; ^*^p < 0.05. The exact *p*-values, from left to right, are as follows: 0.0009 and 0.0081. **(B)** Effect of FMRpolyG on lamin architecture. HEK293 cells were transfected with the indicated constructs and stained for Lamin B1 24 h after transfection. Transfection with GFP-C1 served as negative control. Cells were analyzed by confocal imaging, and the fraction of cells with disrupted lamin architecture counted in GFP positive cells (left) and in cells with aggregates (right). The number of GFP positive cells included in the analysis was >440 for GFP-C1 and wtHP-99Gly-GFP. For wtHP-99Gly-GFP > 170 aggregate bearing cells were counted. Due to the low levels of GFP positive cells among those transfected with mutHP-90Gly-GFP, a total of 80 GFP-positive cells and 70 aggregate bearing cells were included from mutHP-90Gly-GFP transfected cell populations. The exact *p*-values, from left to right, are as follows: 0.0021 (^**^), 0.0004 (^***^), 0.0123 (^*^), and 0.0113 (^*^). **(C)** Representative confocal images showing normal lamin rings in cells expressing GFP (GFP-C1), but disrupted lamin architecture in aggregate-containing cells after transfection of wtHP-99Gly-GFP or mutHP-90Gly-GFP. **(D)** Effect of stably expressed FMRpolyG on lamin architecture. Expression of GFP or FMRpolyG-GFP were induced with doxycycline for 72 h, followed by Lamin B1 staining, confocal imaging, and counting of GFP positive cells with disrupted lamin architecture. Cell expressing GFP (from GFP-C1) were used as negative controls. For GFP-C1 and wtHP-99Gly-GFP, a minimum of 830 GFP positive cells were quantified. Due to low expression levels in cells expressing mutHP-90Gly-GFP, 150 GFP-positive cells were included for this construct. For wtHP-99Gly-GFP and mutHP-90Gly-GFP, a minimum of 80 aggregate bearing cells were analyzed per construct. The exact *p*-values, from left to right, are as follows: 0.0186 (^*^), 0.0007 (^***^), 0.00001 (^***^), and 0.0004 (^***^). **(E)** Representative confocal images showing normal lamin rings in HEK-FlpIn cells expressing GFP and disrupted lamin structures in aggregate-containing cells expressing FMRpolyG-GFP. All graphs in **(A,B,D)** are based on quantifications of a minimum of three individual experiments and error bars represent SD.

Another important histopathological finding in FXTAS patients is the loss of both neuronal and glia cells (Greco et al., 2006). Accordingly, expression of the FMR1-5′UTR including a premutation, has been proven to cause cell death in cultured cells and animal models (Jin et al., 2003; Arocena et al., 2005; Entezam et al., 2007; Hoem et al., 2011). We sought to answer whether expression of the mutHP-90Gly-GFP construct causes cell death. For this purpose, the effect of the FMRpolyG protein on cell viability was assessed by flow cytometry of cells transiently transfected with the wtHP and mutHP constructs in parallel with the vector control GFP-C1. Loss of viability was determined by quantifying the portion of GFP-positive cells which incorporated propidium iodide (PI). Interestingly, we saw no difference in cell viability for the cells expressing FMRpolyG-GFP and RNA hairpin, vs. those only expressing the FMRpolyG-GFP protein (Figure 5A). At 24 h post transfection they both demonstrated ~8% reduction in cell viability, compared to 4% in the GFP-C1-positive control population (Figure 5A). These findings point toward a toxic effect mediated by the FMRpolyG protein, independent from the CGG RNA hairpin formation *per se*.

In several model systems for FXTAS, abnormal lamin ring structure in the presence of FMR1 5′UTR with the CGG-expansion has been reported (Arocena et al., 2005; Hoem et al., 2011; Sellier et al., 2017). Lamin is also present in inclusion bodies present in tissues from FXTAS-patients (Iwahashi et al., 2006). We therefore asked whether the FMRpolyG protein, in the absence of a CGG mRNA hairpin, would affect lamin architecture. To answer this, cells were transfected with wtHP-99Gly-GFP or mutHP-90Gly-GFP, fixed 24 h later and stained with lamin B1 antibody. The number of disrupted lamin rings were quantified in GFP-positive cells and aggregate-bearing cells (Figure 5B). Clearly, there is a strong correlation between the expression of FMRpolyG and observation of disrupted lamin rings, with FMRpolyG-GFP positive cells demonstrating a significant higher portion of abnormal lamin rings compared to GFP-C1 positive controls (Figures 5B,C). Furthermore, cells stably expressing the FMRpolyG protein plus the RNA hairpin did not have a higher portion of disrupted lamin rings compared to those expressing the FMRpolyG protein only.

A similar pattern of lamin ring disruption was observed in HEK-FlpIn cells stably expressing wtHP-99Gly-GFP or mutHP-90Gly-GFP for 72 h. The control was a HEK-FlpIn-GFP-C1 cell line previously described (Larsen et al., 2010) (Figures 5D,E). This demonstrates that the FMRpolyG protein itself is sufficient to impair the lamin structure in cells.

### FMRpolyG Is Immobilized in Aggregates and These Structures Do Not Co-localize With the Microtubule Organizing Center (MTOC)

**Figure 6:**
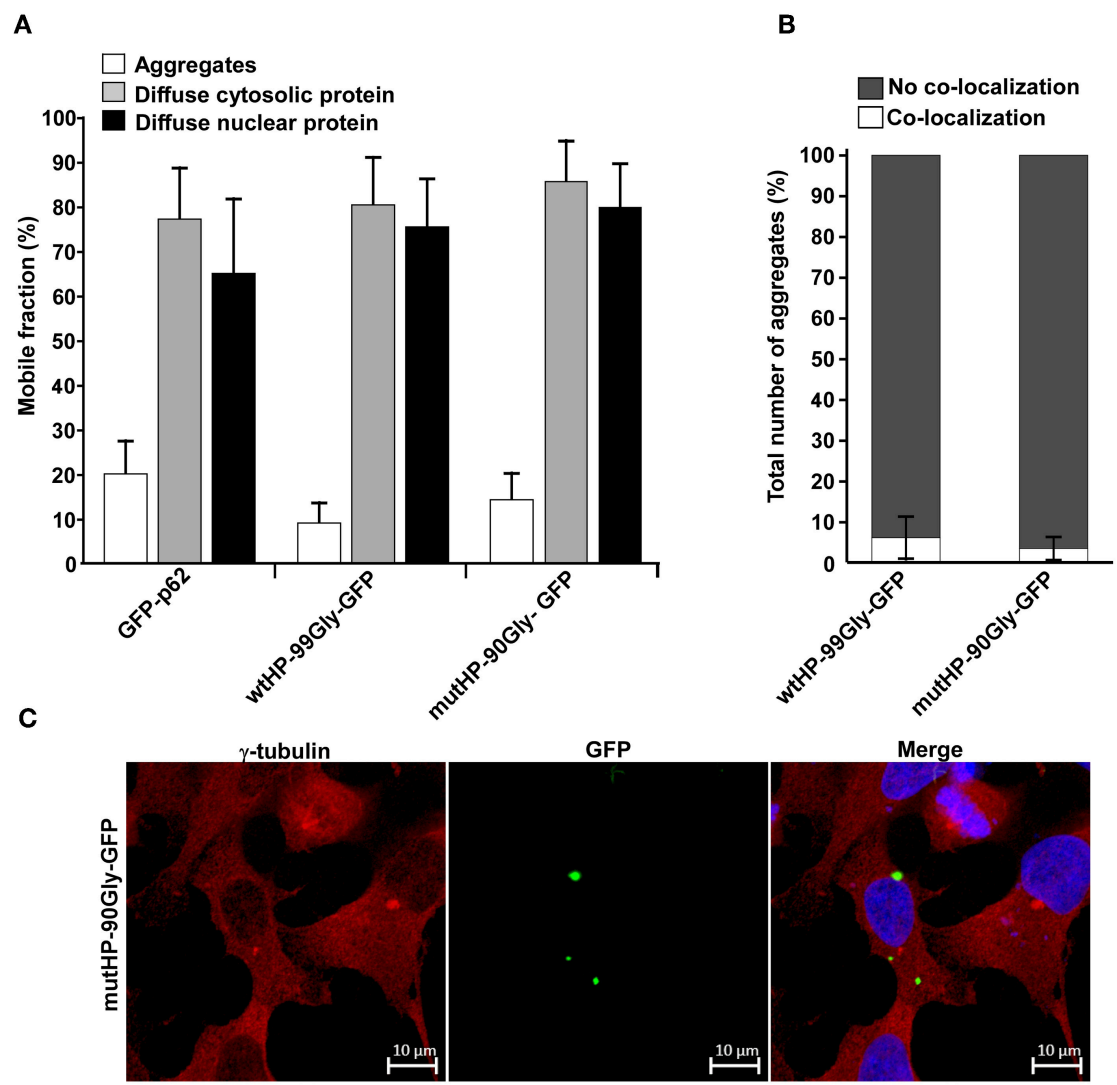
FMRpolyG is immobilized in aggregates and these structures do not co-localize with the microtubule organizing center (MTOC). **(A)** FMRpolyG-GFP is immobile in aggregates. Mobility of FMRpolyG-GFP and GFP-p62 in aggregates and diffuse fractions were analyzed by fluorescence recovery after photobleaching (FRAP). The mobile fractions were calculated using the ZEN 2012 (black edition) software. FMRpolyG-GFP was compared to GFP-p62 known to become immobilized in aggregates. GFP-p62 thus served as a positive control for immobilization in aggregates. Averages are based on a minimum of 8 nuclear foci, 12 cytoplasmic foci, and 12 aggregates for each construct. **(B)** Fraction of FMRpolyG-GFP positive aggregates that co-localize with MTOC. Counting is based on confocal images. The graphs represent three independent experiments, and >60 aggregates per construct. **(C)** Representative confocal image of HEK293 cell with FMRpolyG-GFP aggregates, none of which co-localize with MTOC (red dots). Cells were stained with anti-γ-tubulin antibody to visualize MTOC. Error bars in **(A,B)** represent SD.

Having observed that expression of the FMRpolyG protein alone reduced cell viability, disrupted lamin architecture and formed intranuclear aggregates, we next investigated the protein's mobility in the cell. For this purpose, we applied FRAP (Fluorescence Recovery After Photobleaching) on live cells and studied both the diffuse cytosolic and nuclear portion of the protein, and the aggregate-located portion. We compared the FMRpolyG-GFP expression constructs to GFP-tagged p62. The p62 protein is a well-characterized receptor for selective autophagy (reviewed in Lamark et al., 2009; Khaminets et al., 2016), which has been demonstrated to become quite immobilized in large p62 bodies (Matsumoto et al., 2011). Furthermore, its diffuse soluble fraction is mobile, even though it does to some extent form complexes (Kraft et al., 2016). The p62-protein and its properties has been well-characterized in our lab, and it thus serves as a good reference protein. The FRAP-assay revealed that the fraction of GFP-tagged FMRpolyG protein which is mobile (replaced after photobleaching), is high (>75%) when it appears as diffuse in the cytosol or the nucleus, but very low (<10–20%) in the aggregates (Figure 6A). Importantly, the mobility of these fractions does not differ between wtHP-99Gly-GFP and mutHP-90Gly-GFP, indicating that the dynamics of FMRpolyG is not affected by the CGG mRNA hairpin. In addition, the fractions of GFP-tagged protein which are mobile, are strikingly similar for FMRpolyG and p62. Given p62's known tendency to form aggregates, it is likely that the FMRpolyG protein can behave in a similar way. To conclude, the FRAP-results demonstrate that FMRpolyG is mobile in both the cytoplasmic and nuclear compartments of the cell, but its localization to aggregates affects these dynamics and renders the protein immobile. Importantly, the dynamics of the FMRpolyG protein are independent of the CGG mRNA hairpin.

Next, we asked whether the aggregates themselves were mobile and traveled along microtubuli. To measure this, we quantified co-localization between the microtubule organizing center (MTOC) and the aggregates. No significant co-localization was observed (Figures 6B,C). Moreover, treatment with the microtubule inhibitor nocodazole, did not prevent formation of aggregates (data not shown). Due to the highly toxic effect of long-term nocodazole treatment, we were not able to quantify number of aggregates in this population compared to untreated controls, but aggregates clearly formed in all transfected cell populations. The lack of co-localization with MTOC together with nocodazole's inability to prevent aggregate formation, point toward a microtubule-independent formation of aggregates. Thus, the structures are not classical aggresomes (reviewed in Kopito, 2000).

### FMRpolyG Forms Dense Filamentous Aggregates

**Figure 7:**
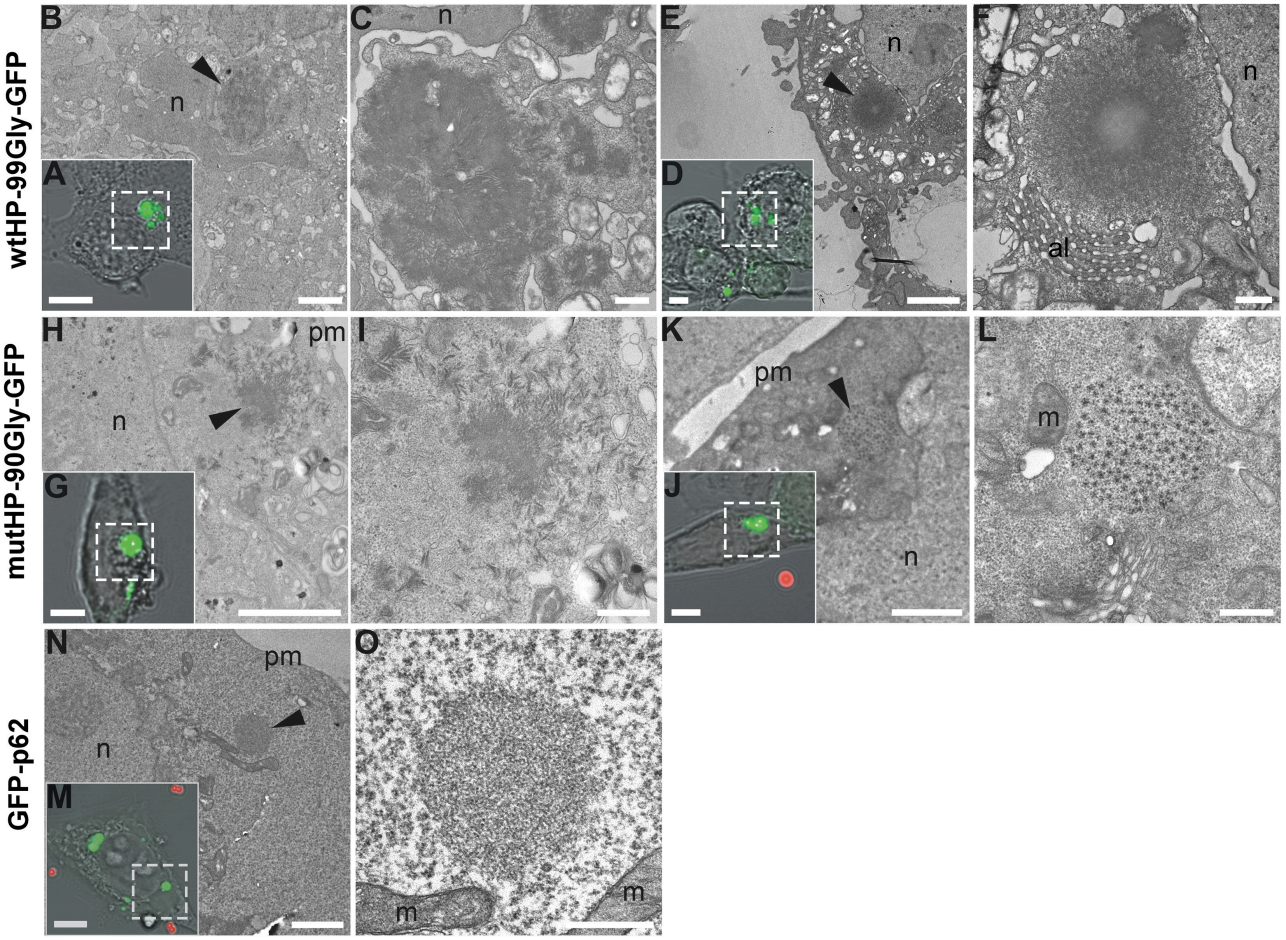
FMRpolyG forms dense filamentous aggregates. Cells expressing GFP-tagged FMRpolyG were fixed and imaged by confocal microscopy to localize aggregate-containing cells, and subsequently processed for transmission electron microscopy. Following ultramicrotomy, cells of interest were relocalized within sections and imaged at both intermediate and high magnifications to analyze the surrounding cellular environment and ultrastructural details of the aggregates, respectively. Transmission electron micrographs of boxed areas in confocal images **(A,D,G,J,M)** are shown at intermediate **(B,E,H,K,N)** and high **(C,F,I,L,O)** magnification. Arrow heads indicate the position of aggregates inside cells. **(A–F)** wtHP-99Gly-GFP aggregates consist of filaments that can be found in parallel orientation to the plane of sectioning over significant lengths (>100 nm), and that lay in close vicinity to the nucleus and to annulate lamellae in ultrathin sections. Note decreased staining toward the center of a large aggregate **(F)**. **(H–L)** mutHP-90Gly-GFP aggregates are similar to wtHP-99Gly-GFP aggregates but appear less densely packed and displayed uniform staining throughout the aggregates in all observed cases. A subset of mutHP-90Gly-GFP aggregates show a highly repetitive pattern of low-contrast spots circularly arranged around a central spot of slightly larger size and higher contrast **(L)**. **(N,O)** In comparison to FMRpolyG aggregates, GFP-p62 aggregates appear to consist of thinner filaments that radiate in all directions within the plane of sectioning. Scale bars are 5 μm **(A,D,G,J,M)**, 2 μm **(B,E,H,K,N)**, and 500 nm **(C,F,I,L,O)**. n, nucleus; al, annulate lamellae; pm, plasma membrane; m, mitochondria. Red spheres in **(J,M)** are fiducial beads used for image registration.

FXTAS inclusions have been examined by electron microscopy and described by both Greco et al. (2002) and Gokden et al. (2009), as compact and granulofilamentous. In order to determine the ultrastructure of the aggregates formed in the cellular systems described here, we performed CLEM (Correlative Light and Electron Microscopy) analyses of both mutHP-90Gly-GFP and wtHP-99Gly-GFP generated aggregates (Figures 7A–L). The ultrastructure of these aggregates was compared to that of aggregates formed by GFP-p62. p62 aggregates have been subjected to CLEM/EM and described as electron dense and with a filamentous appearance (Bjorkoy et al., 2005; Paine et al., 2005; Filimonenko et al., 2007; Nezis et al., 2008; Sukseree et al., 2018). Figures 7M–O show a GFP-p62 aggregate presenting this morphology. Both wtHP-99Gly-GFP and mutHP-90Gly-GFP form dense, filamentous aggregates (Figures 7A–L). The filamentous nature of the aggregates was clearly seen on EM tomography of a wtHP-99Gly-GFP aggregate (Supplementary Video 3). Some of the larger aggregates, primarily seen in wtHP-99Gly-GFP-expressing cells, appear very dense and symmetric, with a center that is brighter (Figure 7F), likely due to incomplete penetration of the heavy metal contrast agents used. Interestingly, some of the mutHP-90Gly-GFP-aggregates are less dense and more granular (Figure 7L). These may represent an earlier, less protein-rich stage of the same aggregates or a different form of aggregates. Notably, these aggregates are generally smaller than the very dense ones. In concordance with observations by Greco et al. (2002) and Gokden et al. (2009), we generally did not see any membrane surrounding the entire aggregates.

### Proteasomes Are Recruited to FMRpolyG Aggregates

**Figure 8:**
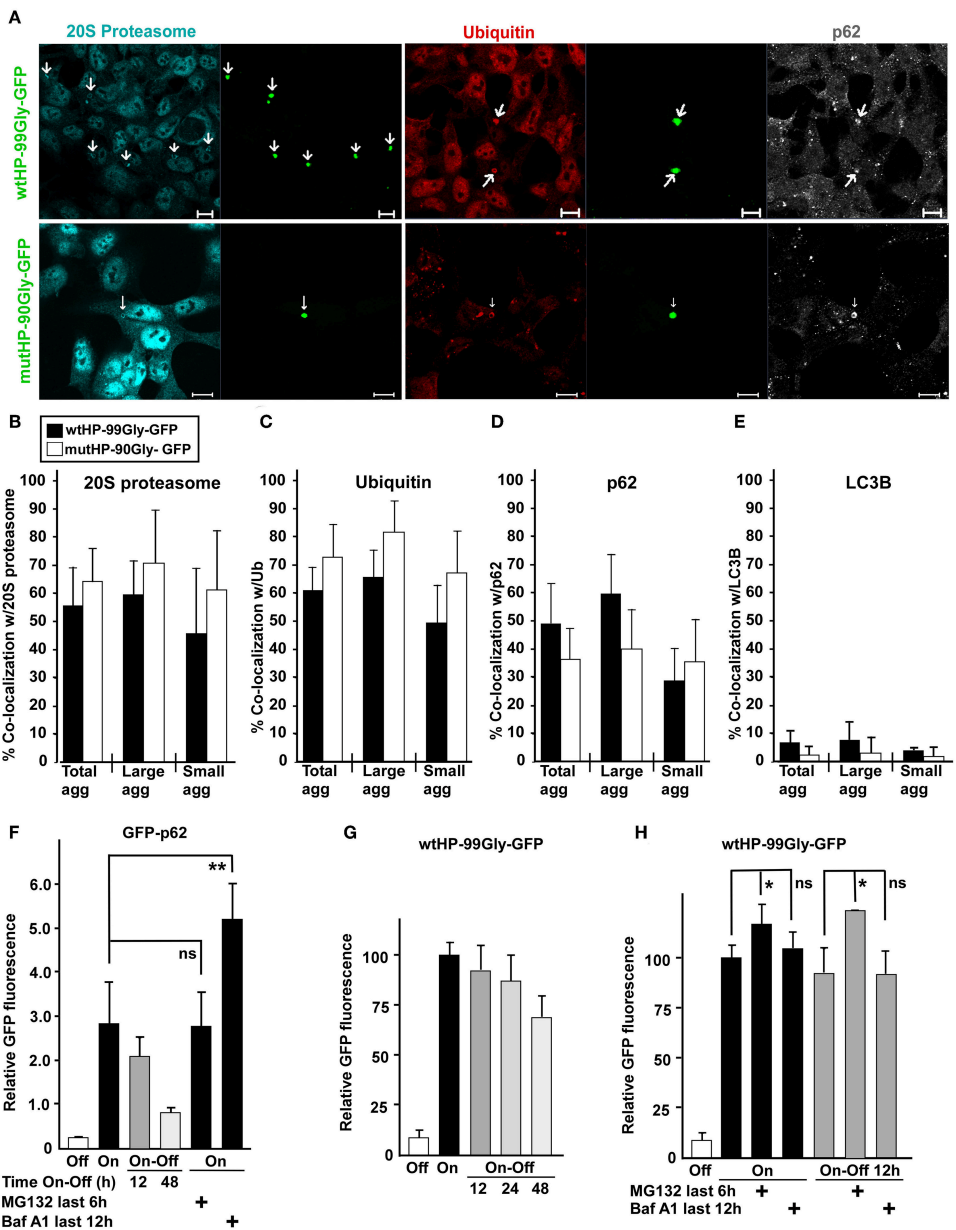
Proteasomes are recruited to FMRpolyG aggregates. **(A)** Representative confocal fluorescence microscopy images of HEK293 cells transfected with wtHP-99Gly-GFP (upper panel) or mutHP-90Gly-GFP (lower panel) and immunostained with antibodies to the proteasome, ubiquitin and p62. Fraction of FMRpolyG-GFP aggregates which co-localized with the proteasome **(B)**, ubiquitin **(C)**, p62 **(D)**, or LC3B **(E)**, after transfection of wtHP-99Gly-GFP (black bars) or mutHP-90Gly-GFP (white bars). Cells were stained for the indicated endogenous proteins. Quantifications were performed using the image analyzing software Volocity, and are based on 3–6 experiments. For **(B)** the total number of aggregates included in the quantification was >65 per construct. The remaining graphs **(C–E)** are based on analysis of a total of > 190 GFP-positive aggregates per construct. **(F–H)** FMRpolyG is mainly degraded by the proteasome. Except for the negative controls (uninduced cells), HEK-FlpIn cells were treated with tetracycline (1 μg/ml) for 48 h to induce accumulation of GFP-p62 **(F)** or FMRpolyG-GFP **(G,H)**, respectively. Degradation was then measured by flow cytometry of the entire cell population (>20,000 cells for each condition, per experiment), as a loss in mean GFP intensity after the removal of tetracycline (Tet Off). The experiments were performed as indicated in the absence or presence of Baf-A1 or MG132. All graphs are based on a minimum of three independent experiments. The exact *p*-values in **(F)** are 0.67 (for the n.s. difference), and 0.006 (for difference marked by two stars). The exact *p*-values, from left to right, in **(H)** are as follows: 0.029 (one star), 0.402 (n.s.), 0.048 (one star), and 0.933 (n.s.). Error bars represent SD.

Here we have shown that the FMRpolyG protein, even in the absence of a CGG RNA hairpin, forms dense aggregates and has a toxic effect on cells. Degradation of proteins that are misfolded, toxic or for other reasons labeled as unwanted by the cell, is carried out by autophagy and/or the ubiquitin-proteasomal system (UPS). Studies performed in a *Drosophila* model for FXTAS have demonstrated that inhibition of UPS increases neurodegeneration, while inhibiting autophagy can improve the phenotype (Oh et al., 2015). Moreover, mayor players in the UPS, namely ubiquitin and the proteasome, are present in FXTAS inclusions (Iwahashi et al., 2006; Lin et al., 2013). With this in mind, we asked whether protein components of the UPS and/or the autophagy machinery co-localized with FMRpolyG-aggregates in our system. For this purpose, cells containing FMRpolyG aggregates were stained with antibodies to marker proteins for UPS (20S proteasome and ubiquitin) and autophagy (LC3B and p62), and analyzed by fluorescence confocal microscopy. The majority of aggregates contained both ubiquitin and the 20S proteasome (Figures 8A–C). Interestingly, p62, an autophagy receptor involved in both autophagic and proteasomal degradation of proteins (Pankiv et al., 2007; Geetha et al., 2008), was enriched in ~35–50% of the aggregates (Figures 8A,D). p62 has previously been found in FXTAS-inclusions (De Pablo-Fernandez et al., 2015). In contrast, LC3B, a major adaptor and marker in the autophagy pathway, was not found to be present in the aggregates (Figure 8E). Importantly, we find the numbers of p62-, proteasome-, and ubiquitin positive aggregates to be similar in wtHP-99Gly-GFP and mutHP-90Gly-GFP expressing cells.

### Degradation of the FMRpolyG Protein Is a Slow Process Impeded by UPS Inhibition

The presence of ubiquitin and the 20S proteasome in FMRpolyG aggregates, prompted us to investigate how the FMRpolyG protein is degraded. Since the FMRpolyG protein has demonstrated the same tendency to form aggregates, cause cellular toxicity, has the same mobility, and co-localizes with the same proteins whether it is produced from wtHP-99Gly-GFP or mutHP-90Gly-GFP, we chose the HEK-FlpIn cell line with the highest expression of the FMRpolyG protein (wtHP-99Gly-GFP) for degradation studies. Using a flow-based assay, we monitored GFP-fluorescence in a “tet-on-off” experiment. In parallel, we followed the degradation of GFP-p62 in a HEK-FlpIn cell line (Larsen et al., 2010). The GFP-p62-protein has a half-life of ~14 h in full medium in HEK cells (Larsen et al., 2010), and is degraded through autophagy (Bjorkoy et al., 2005; Pankiv et al., 2007).

By adding tetracycline, expression of GFP-fusion proteins in the cells was first induced for 48 h, followed by 12, 24, or 48 h in tet-free medium (tet-off). This allows monitoring of how the GFP-tagged FMRpolyG protein-levels decrease when expression is turned off. In parallel, cells were treated with either the proteasomal inhibitor MG132, or the lysosomal inhibitor Bafilomycin A1 (BAF A1). GFP-p62-levels were reduced by more than 70% after 48 h with tet-off. As expected, GFP-p62 accumulated upon BAF-A1 treatment, while its levels were unaltered by inhibiting the proteasome with MG132 (Figure 8F). In contrast, FMRpolyG was degraded slowly, with barely a 30% reduction of GFP-levels after 48 h with tet-off (Figure 8G). Furthermore, FMRpolyG-levels increased upon inhibition of the proteasome, while treatment with the lysosomal inhibitor had a minor impact (Figure 8H). These observations indicate that FMRpolyG is a very stable protein which is degraded primarily through the UPS. The results are consistent with a model where very low expression levels of FMRpolyG can accumulate over time and cause toxicity late in life.

## Discussion

Numerous studies have demonstrated a toxic effect by expressing mRNA harboring an expanded CGG repeat sequence (Jin et al., 2003; Willemsen et al., 2003; Arocena et al., 2005; Entezam et al., 2007; Hashem et al., 2009; Hoem et al., 2011; Hukema et al., 2014). The finding of RAN-translation from the expanded CGGs (Todd et al., 2013), hypothesized that the RAN-translation product FMRpolyG contributes to this toxicity. Sellier et al. (2017) have recently shown that expression of the FMRpolyG protein together with the CGG mRNA hairpin, is more toxic than expressing the CGG mRNA alone, and that the C-terminal end of FMRpolyG reduces cell viability when expressed without the remaining protein sequence. Previous studies have not investigated the effects of expressing the complete FMRpolyG protein without the CGG mRNA hairpin.

We have established a cellular model where the FMRpolyG protein is expressed without the corresponding CGG mRNA hairpin. This allows analyses of the effects mediated by the FMRpolyG protein without any CGG mRNA. Using this system, we are, to our knowledge, the first to show that the complete FMRpolyG protein causes aggregate formation across several cell lines, forms intranuclear aggregates, reduces cell viability and disrupts the architecture of lamin rings, in the absence of CGG mRNA. Furthermore, expression of the FMRpolyG protein together with the native RNA-hairpin-forming CGG repeats did not increase features of cellular toxicity such as cell death or lamin ring disruption, compared to that observed with the FMRpolyG translated from mRNA without CGG repeats.

Notably, a parallel can be drawn to studies of RAN-translated dipeptide repeat proteins (DRPs) arising from the chromosome 9 open reading frame 72 (C9ORF72) G_4_C_2_-expansion causing amyotrophic lateral sclerosis and frontotemporal dementia (ALS-FTD, #105550). Expression of synthetic DRPs where the G_4_C_2_ repeats have been substituted by alternative codons for the same peptide sequence, results in toxicity and aggregate formation (May et al., 2014; Mizielinska et al., 2014; Yamakawa et al., 2015). This, together with findings both in the current study and by Sellier et al. (2017), suggest that toxicity mediated by expression of the peptide sequence of RAN translated proteins is a general mechanism that may occur in several repeat associated disorders.

In our model, substituting the CGG repeats with alternative codons for the polyGlycine stretch in the FMRpolyG protein, resulted in a several-fold reduced expression of both mRNA and protein from the gene. This supports an already proposed *cis*-acting effect on transcription mediated by the CGGs themselves (Chen et al., 2003). Because GGC is the most frequently used glycine codon, we cannot exclude that the change into less common glycine codons, contribute to the low expression from the muHP construct by reducing translation efficiency. However, there is also much less mRNA produced from the mutHP construct, supporting that the main difference is at the level of transcription. It is well-known that the FMR1 mRNA levels in PM carriers are 2–8-folds higher than observed for non-expanded alleles (Tassone et al., 2000). This is close to the ~11-fold difference we observed between the wtHP-99Gly-GFP and the mutHP-90Gly-GFP mRNA levels we observed in the HEK-FlpIn cell lines. Elevated mRNA levels from expanded alleles are also found in mice (Entezam et al., 2007; Brouwer et al., 2008) and upon transient transfection of HEK293 cells (Chen et al., 2003). The reason for the elevated mRNA levels from premutation alleles appears to be increased transcription rather than mRNA stability (Tassone et al., 2007).

Our findings show that FMRpolyG, in the absence of CGG mRNA, causes both cell death and lamin ring disruption, two important features of cellular toxicity observed in previous model systems for FXTAS (Arocena et al., 2005; Hoem et al., 2011; Sellier et al., 2017). This is consistent with Sellier et al.'s (2017) observations that the C-terminal end of the FMRpolyG was required for lamin ring disruption and played an important role in reducing cell viability. However, toxicity is also observed in a mouse model for FXTAS where the FMRpolyG protein is not expressed (Entezam et al., 2007; Todd et al., 2013). This suggests that the CGG mRNA has an independent toxic effect that does not rely on FMRpolyG expression. In our study, we do not find increased toxicity for FMRpolyG plus CGG mRNA compared to FMRpolyG alone, but the CGGs have a strong impact on the expression level of the FMRpolyG. A toxic effect of the FMRpolyG protein can thus be interpreted as further evidence for the CGG's importance in triggering FXTAS.

The C-terminal end of FMRpolyG, which mediates the protein's toxic effect (Sellier et al., 2017), is identical in both non-affected individuals with a normal allele and FXTAS-patients with the CGG repeat expansion. It is the number of CGGs in the FMR1 gene that differs. The expanded CGG repeat tract's role in increasing FMRpolyG levels, and thus amount of the toxic C-terminus, may therefore be an important factor in triggering the FXTAS pathogenesis. It is worth noting that lamin ring disruption and cell death are also observed in both an episome based cellular model system and upon transient transfection of cultured neuronal cells, where only the first part of FMRpolyG's C-terminal sequence is present (Arocena et al., 2005; Hoem et al., 2011). Thus, even though FMRpolyG expression is toxic, it appears that the protein's very C-terminal end is not required for lamin ring disruption or reduction of cell viability. Further studies are needed to determine the exact C-terminal sequence mediating these effects.

In addition to toxicity, our study has revealed that FMRpolyG has capability to induce formation of protein aggregates, without the presence of CGG mRNA. We observed both nuclear and cytoplasmic aggregates. While cytoplasmic aggregates are prevalent in a *Drosophila* model of FXTAS (Jin et al., 2007), patient material reveal inclusions exclusively in the nucleus (Greco et al., 2002; Hunsaker et al., 2011). We therefore cannot exclude that formation of intranuclear aggregates in patients arise through other pathways than the aggregates observed in this study, and in the *Drosophila* model. Nonetheless, our main finding concerning aggregate formation is that presence or absence of the CGG mRNA does not affect aggregate formation, localization or mobility. In addition, we have applied electron microscopy to reveal that the ultrastructure of these aggregates is mainly filamentous, dense and non-membrane bound. Importantly, inclusions in FXTAS patients are reported to have similar morphological features (Greco et al., 2002; Gokden et al., 2009). This is to our knowledge the first study of the ultrastructure of FMRpolyG-induced aggregates. Interestingly, polyGlycineAlanine (poly-GA) aggregates have recently been studied using cryoelectron tomography (Guo et al., 2018). This dipeptide is part of a protein produced by RAN translation across the G_4_C_2_ repeats in C9ORF72 ALS/FTD. The authors show that poly-GA aggregates recruit the proteasomes (Guo et al., 2018). Since the FMRpolyG aggregates stain positive for the 20S proteasome, it is possible that the glycine in both poly-GA and FMRpolyG aggregates interacts directly with the proteasome to mediate this sequestration.

Finally, our study is the first to assess important features of the FMRpolyG protein such as its mobility in different cellular compartments and the rate and pathway for its degradation. We show that the FMRpolyG is a stable protein, primarily degraded through the UPS. Interestingly, a previous study has pointed out that inhibiting the UPS leads to increased neurodegeneration in FXTAS model systems (Oh et al., 2015). Moreover, this effect appears to be dependent on FMRpolyG expression (Oh et al., 2015). Our study not only supports the notion that increased level of FMRpolyG is involved, but also provides evidence for degradation of the FMRpolyG through the UPS. Targeting this pathway thus appears to be an option for therapeutic intervention that should be further explored.

In conclusion, the current study not only provides further evidence for FMRpolyG's possible role in causing cellular dysregulation, but it demonstrates for the first time that expression of the complete FMRpolyG protein, without the presence of the CGG mRNA hairpin, is sufficient to form aggregates and cause different features of cellular toxicity. This is a proof of principle regarding toxicity of the FMRpolyG protein, demonstrating that the specific CGG-repeat sequence is not required for this effect. However, several questions regarding FMRpolyG's exact role in the pathogenesis of FXTAS still remain open. Specifically, no functional domains or motifs of the FMRpolyG protein are described, and how it mediates inhibition of the UPS has not been elucidated. To further determine which cellular features of FXTAS are triggered by the CGG mRNA and the FMRpolyG, respectively, studies focusing on endogenous expression level in patient materials are warranted. Addressing these issues is crucial to understand the role of FMRpolyG in the pathogenesis of FXTAS and will hopefully bring us closer to targeted treatments.

## Materials and Methods

### Cell Culture and Transfection

All cell lines were grown in DMEM (Sigma-Aldrich, D6046) supplemented with 10% Fetal Bovine Serum (FBS) (Biochrom, S0615) and 1% streptomycin-penicillin (Sigma-Aldrich, P4333) at 37°C and 5% CO_2_. For immunofluorescence staining and microscopy analysis, subconfluent cells were transfected with plasmids using Trans-IT-LT1 (Mirus, MIR2300). For flow cytometry analyses, mRNA quantifications or immunoblotting, cells were transfected using Metafectene Pro (Biontex, T040-1.0). All transfections were performed according to the respective manufacturer's protocols.

### Plasmids

The wtHP-99Gly-GFP-plasmid and a wtHP-ACG-99Gly-GFP-plasmid (Addgene plasmid # 63091) were kind gifts from Nicolas Charlet-Berguand (Sellier et al., 2017). The mutHP-90Gly-GFP plasmid was made in two steps. First a modified version of the coding sequence for the FMRpolyG protein, harboring 90 consecutive glycine codons without the CGG repeat sequence (see sequence comparison in Supplementary Figure 1), was ordered from Thermo Fischer Scientific. The sequence came incorporated in a pDONR221-plasmid. Secondly, the C-terminal GFP-tag was inserted by using the XhoI- and XbaI-sites in both the pDONR221-plasmid and the wtHP-99Gly-GFP plasmid, and then the entire sequence replaced the coding sequence in the wtHP-99Gly-GFP plasmid, using the DraI or NarI, and XbaI restriction sites. The resulting plasmid was named mutatedHairPin-90Glycine-GFP (mutHP-90Gly-GFP). pDEST-GFP-C1 and pDEST-eGFP-p62 have been described previously (Lamark et al., 2003). Due to the inherent instability of the CGG-repeats, all constructs were cut with restriction enzymes and run on agarose gels to ensure that the CGG and GGN-repeat tracts were not deleted or contracted. All constructs were also subject to Sanger sequencing from both C- and N-terminal sides, to ensure that sequences surrounding the repeats were maintained.

### Establishment of Inducible wtHP-99Gly-GFP and mutHP-90Gly-GFP HEK-FlpIn Lines

Flp-In-T-Rex 293 (HEK-FlpIn) cells (Invitrogen, R780-07) were seeded out at approximately 25% confluency in T25 flasks 2 days prior to transfection. In FBS- and antibiotic free medium, 0.5 μg of pcDNA5/FRT/TO- mutHP-90Gly-GFP or pcDNA5/FRT/TO-wtHP-99Gly-GFP and 1.5 μg of pOG44 was added, and subsequently mixed with medium containing 4 μl of Metafectene Pro (Biontex, TO40). The mix was incubated for 20 min before it was added to the cell media in T25 flasks with HEK-FlpIn cells. After 24 h, the medium was changed to regular DMEM with supplements, and the day after that to DMEM with 150–200 μg/ml of hygromycin B (Sigm-Aldrich, 108435550019) and 15 μg/ml of blasticidin (Sigma-aldrich, SBR00022). Cells were kept in this medium for 3–6 days, depending on the number of viable cells, and subsequently in regular DMEM with supplements. The cells were tested for their ability to express the GFP-tagged protein of interest, by treatment with 1 μ/ml of doxycycline for 24–72 h followed by fluorescence microscopy assessing the GFP-expression, and Western Blotting to confirm that the GFP-tagged protein was of the predicted size. Prior to all experiments, cells were maintained 1–2 weeks in complete DMEM with 100 μg/ml of hygromycin B. The FlpIn-GFP and FlpIn GFP-p62 cell lines have been described previously (Larsen et al., 2010).

### Antibodies and Reagents

The following primary antibodies were used: guinea pig anti-p62 (Progen, GP62-C), rabbit anti-Lamin B1 (Abcam, ab16048), rabbit anti-20S proteasome (Enzo Life Science, PW8155), mouse anti-γ-tubulin (Sigma-Aldrich, T6557), mouse anti-ubiquitin (Biomol, PW8810), rabbit anti-LC3B (Novus, NB100-2220), rabbit anti-GFP (abcam, ab290), mouse anti-FLAG (M2) (Sigma-Aldrich, F3165), and mouse anti-β-tubulin (Sigma-Aldrich, T4026). Secondary antibodies used for IF were all Alexa Fluor® antibodies from Life Technologies: 555-conjugated goat anti-mouse (A21424), 555-conjugated goat anti-rabbit (A21429), 555-conjugated goat anti-guinea pig (A21435), 488-conjugated goat anti-mouse (A11029), 647-conjugated goat anti-rabbit (A21245), 647-conjugated goat anti-guinea-pig (A21450), and 647-conjugated anti-mouse (A21236). Secondary antibodies used for western blot were as follows: HRP-linked goat anti-rabbit IgG (554021, BD Pharmingen), HRP-linked goat anti-mouse IgG (554002, BD Pharmingen), Anti-biotin HRP-linked antibody (Cell signaling, 7075P5).

### Real-Time PCR

FlpIn HEK293 cells were induced to express either wtHP-99Gly-GFP, mutHP-90Gly-GFP or GFP-C1 by adding 1 μg/ml of doxycycline for 72 h, and HEK293 cells were transfected 24 h prior to RNA extraction using the GenElute Mammalian Total RNA Miniprep Kit (Sigma-Aldrich). Transcriptor Universal cDNA Master (05893151001, Roche) was used for cDNA synthesis from total RNA. RT-PCR was performed using FastStart Universal SYBR Green Master (Roche) on a LightCycler 96 Real-Time PCR System (Roche). Primers were as follows, for GFP: 5′-ACGTAAACGGCCACAAGTTC−3′ and 5′ -AAGTCGTGCTGCTTCATGTG- 3′, for GAPDH: 5′–GGCACTGTCAAGGCTGAAAACG−3′ and 5′- GGAGATGAGATGATACCACGCTTAG−3′. The ΔΔCt-method (described in Applied Biosystems User Bulletin No. 2 (P/N 4303859)) was used to calculate relative mRNA levels using GAPDH as an internal reference.

### Western Blots and Immunoprecipitation

Cells were harvested in buffer containing 2% SDS, 10% glycerol, and 50 mM Tris-HCl, and immediately boiled for 10 min. Protein concentration was measured using the Pierce BCA Protein Assay Kit (Thermo Fischer Scientific # 23225). DTT (100 mM) and bromophenol blue was added to samples which were run on a 12% SDS-polyacrylamide gel. Blotting was performed on nitrocellulose membranes (GE Healthcare 10600003) which were then stained with Ponceau S. Dry milk (5%) in phosphate buffered saline with 0.1% tween-20 (PBS-T) was used for both blocking of membranes and dilution of secondary- and primary antibodies. Blocking was performed for 30 min at room temperature, incubation with primary antibody at 4°C overnight, and incubation with secondary antibody for 1 h at room temperature. Membranes were washed minimum three times in PBS-T both before addition of secondary antibody, and again before development using a Chemiluminicent Peroxidase substrate kit (Sigma-Aldrich, CPS3500) and an ImageQuant LAS 4000 machine (GE Healthcare).

### Immunofluorescence Staining

Cells were seeded on glass coverslips (VWR, 631-0150) coated with fibronectin (F1141-5MG, Sigma-Aldrich) at 24–48 h prior to transfection or induction of expression. At indicated time points after transfection or induction of expression, cells on coverslips were fixed in 4% formaldehyde (Merck KGaA, 1.00496.5000) for 20 min at RT and washed twice in PBS. Cells were then permeabilized in ice cold MeOH for 9 min and subjected to blocking in 3% goat serum/PBS (GS/PBS) for 30 min at RT. This was followed by incubation with primary antibody (diluted in 1% GS/PBS) for 90 min, 5 washes in PBS, incubation with secondary antibody (diluted in 1% GS/PBS) for 75 min, and 5 new washes in PBS before samples were stained with DAPI (Thermo Scientific, 62248) and washed in PBS and dH_2_O. Finally, the coverslips with stained cells were mounted by use of Mowiol 4–88 (Calbiochem) mounting medium on microscopy slides.

### Flow Cytometry

For cell viability assays, cells were seeded in 6-well plates the day before transfection with either pDEST-GFP-C1, wtHP-99Gly-GFP, or mutHP-90Gly-GFP. Both floating and attached cells were collected 24 h after transfection, pelleted at 2,000 g for 5 min and resuspended in 500 μl of PBS with Propidium Iodide (PI) (Thermo Fischer, P4864) at a final concentration of 50 μg/ml. Thresholds for PI-positive and GFP-positive cells were set by first analyzing samples with untransfected cells in PBS without PI. For FACS analysis of protein degradation, FlpIn HEK293 cells were treated with 1 μg/ml of tetracycline for 48 h, except for negative controls. Wells subjected to “tet-off” treatment were washed twice in tet-free DMEM and maintained in tet-free medium for the times indicated for each well. The inhibitors Bafilomycin A1 (final concentration 200 nM) and MG-132 (final concentration 10 μM) were added to the indicated wells at 6 and 12 h, respectively, before flow analysis. The above mentioned FACS analyses were performed on a BD LSRFortessa machine (BD Biosciences) using the BD FACSDiva software.

To assess the portion of FlipIn HEK293 cells expressing GFP-tagged proteins, cells were seeded in T75 flasks and treated with 1 ug/ml of tetracycline for 24 h. Cells were trypsinized and resuspended in DMEM media immediately before analysis. Sorting and measurement of GFP-intensity was carried out on a BD FACSAria III Flow Cytometer and Cell sorter, using the BD FACSDiva 8.01 Software.

### Confocal Microscopy and Quantifications

For confocal microscopy a Zeiss Axio Observer.Z1 LSM780 system (Carl Zeiss Microscopy GmbH, Germany) was used. Quantifications were performed using the Volocity Software (PerkinElmer).

### Long-Term Imaging of Live Cells

Cells were seeded in 35 mm Ibidi μ-dishes (81156, Ibidi), coated with fibronectin, 2 days before transfection. Cells were transfected with either mutHP-90Gly-GFP or wtHP-99Gly-GFP, and inserted into a pre-warmed BioStation IM-Q Cell-S2 (Nikon) where 10–15 regions of interest were photographed every 4 min for 12–72 h. The resulting films were processed using the Volocity software (PerkinElmer).

### Fluorescence Recovery After Photobleaching (FRAP)

FRAP-experiments were performed 24–48 h after transient transfection of cells. During imaging, cells were maintained in DMEM with supplements, at 37°C and 5% CO_2_. Imaging was performed using the C-Apochromat 40X NA1.2W objective on the Zeiss Axio Observer.Z1 LSM780 system. Using the ZEN 2012 (black edition) software, cells were imaged every 100 msec. At least 50 pre-bleach images were taken before bleaching with the 488-nm laser (at 100%) for 25–50 iterations. Post-bleaching 1,000–3,000 images were captured with 100 ms intervals. For GFP-protein diffuse in the nucleus or cytoplasm, both bleaching and measurements of recovery were performed in circles with a diameter of 5.8 μm. Due to variation in size of aggregates, circles were individually adapted to fit the size of the entire aggregate. Calculation of mobile fraction of the different GFP-positive protein species, was performed using the FRAP function in the ZEN 2012 (black edition) software. Recovery in bleached areas was normalized to both background and an unbleached reference region. The data was fitted to a mono exponential model using the ZEN 2012 FRAP function. From these calculations the mobile fraction was extracted for each bleached area. For all GFP-tagged proteins, analysis of a minimum of 8 areas with diffuse GFP-protein in the nucleus, 12 with diffuse GFP-protein in the cytoplasm, and 12 GFP-positive aggregates from more than three experiments were combined to create averages and standard deviation (SD) of mobile fractions.

### Correlative Light and Electron Microscopy (CLEM)

Cells were seeded on glass-bottom dishes (Mattek cat. no. P35G-1.5-14-CGRD) with an engraved grid pattern to permit correlation of dish coordinates between light microscope images and the resin block face prior to ultramicrotomy. Cells were transfected with wtHP-99Gly-GFP or mutHP-90Gly-GFP 2 days after seeding, as previously described. The day after transfection, cells were fixed in pre-warmed (37°C) fixative containing 4% formaldehyde, 0.5% glutaraldehyde, and 0.05% malachite green in PHEM buffer (60 mM PIPES, 25 mM HEPES, 10 mM EGTA, 4 mM MgSO_4_·7H_2_O) using a microwave processor (Ted Pella, Inc.). Cells were then imaged by confocal microscopy as previously described. After confocal microscopy, cells were further microwave-processed using additional fixatives and contrast agents for electron microscopy: 1% osmium tetroxide/0.8% K_3_Fe(CN)_6_ in PHEM buffer, 1% tannic acid in ddH_2_O, and 1% uranyl acetate in ddH_2_O. Finally, samples went through stepwise ethanol dehydration (30-60-96-100%) and embedding in EPON resin. The resin was polymerized at 60°C for 48 h and then trimmed to the relevant dish coordinate. Seventy nanometer serial sections were cut using a diamond knife (Diatome) on a Reichert Ultracut S ultramicrotome (Leica) and picked up on formvar-coated slot grids. Sections were imaged using a JEM-1010 transmission electron microscope (JEOL) equipped with a Morada CCD camera and iTEM software (Olympus). Subsequent image processing (stitching, overlay, etc.) was performed using Photoshop (Adobe).

For electron tomography of CLEM-samples we prepared sections of 200 nm that were placed on formvar-coated slot grids and incubated with 10 nm protein A-gold on both sides (2 min each) as fiducials. Dual-axes tilt series were obtained in a Thermo ScientificTM TalosTM F200C microscope at 200 kV, with image series taken at tilt angles from −60 and 60 and 2° increment with pixel size of 0.84 nm. Images were recorded with a Ceta 16M camera and tomograms reconstructed by weighted back projection using IMOD software (version 4.9). The software was also used for movie montage and final movie clips were generated using Fiji software.

### Statistics

All experiments were repeated at least three times, unless otherwise specified. Error bars represent the standard deviation, and two-sided Student *t*-tests were performed to assess significant differences between populations. Replicates were not pooled for statistical analyses. One star (^*^) denotes *P* < 0.05, two stars (^**^) *P* < 0.01, and three stars (^***^) *P* < 0.001.

## Data Availability

All datasets generated for this study are included in the manuscript and/or the Supplementary Files.

## Author Contributions

GH, TL, ES, and TJ designed the study. GH, KB, ES, TL, and TJ prepared the figures. Experiments were performed by GH, AØ, KB, AB, and ES. GH, ES, TL, and TJ wrote the manuscript.

### Conflict of Interest Statement

The authors declare that the research was conducted in the absence of any commercial or financial relationships that could be construed as a potential conflict of interest.
