# Supplementary material for: The FMRpolyGlycine Protein Mediates Aggregate Formation and Toxicity Independent of the CGG mRNA Hairpin in a Cellular Model for FXTAS
Source: Front Genet. 2019 Mar 28;10:249. doi: 10.3389/fgene.2019.00249 (PMC6447689; doi:10.3389/fgene.2019.00249)

The FMRpolyGlycine protein mediates aggregate formation and toxicity independent of the CGG mRNA hairpin in a cellular model for FXTAS

Supplementary Material

Figure legends supplementary figures

Supplemental Figure 1. (**A**) Alignment of the nucleotide sequences of the wtHP-99Gly-GFP and mutHP-90Gly-GFP constructs. The start codon for FMRpolyG-GFP is indicated in yellow. There is a difference in the region 5' to the FMRpolyG encoding region, but this does not affect the sequence of FMRpolyG proteins produced. CGG repeat codons of wtHP-99Gly are indicated in red, and EGFP codons in green. (**B**) Alignment of wtHP-99Gly-GFP with an alternative mutHP-90Gly-GFP construct with a 5' region 100% identical to that of wtHP-99Gly-GFP. These sequences are identical, except for the substitution of the CGGs by alternative Gly codons. Analysis of the sequences using «Rare Codon Analysis Tool» from genescript (https://www.genscript.com/tools/rare-codon-analysis), show that they may both impair translation. The mutHP sequence has a codon adaptation index (CAI) of 0.66 vs 0.89 for the wtHP sequence. This could indicate greater translation inhibition due to less frequent use of the most common glycine codon (GGC) for the mutHP sequence. However, both GC-content and number of negative repeat elements is higher for the wtHP sequence, and the percentage of low frequency (<30%) codons is 3% for both.

Supplemental Figure 2. (A) Predicted secondary mRNA structures of the polyGlycine coding regions of wtHP-99Gly and mutHP-90Gly RNA, based on RNAfold. The CGG repeats in wtHP-99Gly are predicted to form a very strong hairpin structure while the alternative codons in mutHP-90Gly RNA are not predicted to form any stable secondary structure. Note the differences in free energy and frequency of the minimal free energy (MFE) structures. (B and C) Confocal images of HEK 293 cells expressing constructs where the atg has been replaced with the native acg. (**B**) HEK 293 cells imaged 48 hrs after transfection with **acg**-wtHP-99Gly-GFP. FMRpolyG aggregate in the center of the picture (**C**) HEK 293 cells imaged 48 hrs after transfection with **acg**-mutHP-99Gly-GFP

Supplemental Figure 3. Variations 5' to FMRpolyG coding sequences do not explain differences in transcription level. HEK293 cells were transfected with the indicated constructs and analyzed for GFP mRNA levels (upper panel) and FMRpolyG-GFP aggregate formation (lower panel). Imaging and harvesting of RNA was performed on HEK293 cells 24 hours after transfection. The "new mutHP-90Gly-GFP" construct has the exact same coding sequence as mutHP-90Gly-GFP, but the part of the 5'UTR which differs between mutHP-90Gly-GFP and

wtHP-99Gly-GFP, is modified to be exactly the same as in the wtHP-99Gly-GFP (see supplemental figure 1). Changing this part of the 5'UTR did not cause increase in mRNA-levels (upper panel) or the ability to form aggregates (lower panel), indicating that it is the CGGs themselves that cause increased levels of transcript from wtHP-99Gly-GFP. White arrowheads in confocal images indicate GFP-positive aggregates.

Supplemental Figure 4. Differences in mRNA and protein levels in whole cell populations stably or transiently transfected with wtHP-99Gly-GFP or mutHP-90Gly-GFP. (A-C) Data from HEK 293 cells transiently transfected. (D-E) Data from HEK-FlpIn cells stably expressing wtHP-99Gly-GFP or mutHP-90Gly-GFP. (**A**) qPCR data showing average fold difference in GFP-mRNA levels between cells transfected with wtHP-99Gly-GFP or mutHP-90Gly-GFP. The graph represents the average of five individual experiments. (**B**) Western blot of FMRpolyG-GFP in HEK293 cell lysates harvested 24 hours after transfection with wtHP-99Gly-GFP or mutHP-90Gly-GFP. The blots were stained as indicated with antibodies against GFP and β-tubulin. (**C**) The graph shows fold difference in FMRpolyG-GFP protein levels in cells transfected with wtHP-99Gly-GFP and mutHP-90Gly-GFP. GFP-positive bands were normalized to β-tubulin and quantified using ImageJ (**D**) qPCR data showing average fold difference in GFP-mRNA levels between HEK-FlpIn cells stably expressing wtHP-99Gly-GFP or mutHP-90Gly-GFP, after induction with doxycycline for 24 hours. (**E**) Western blot of FMRpolyG-GFP in lysates from HEK-FlpIn cells stably expressing wtHP-99Gly-GFP or mutHP-90Gly-GFP. Lysates were harvested after induction of FMRpolyG-GFP expression with doxycycline for 72 hours. (**F**) The graph shows fold difference in FMRpolyG-GFP protein levels in cells expressing wtHP-99Gly-GFP or mutHP-90Gly-GFP. GFP-positive bands were normalized to β-tubulin and quantified using ImageJ. Error bars in A, C, D and F represent SD of averages from a minimum of three independent experiments.

Supplemental Video 1. wtHP-99Gly-GFP-transfected cells start to form the first aggregates 7 hours after transfection. Long-term imaging of live HEK293 cells transfected with wtHP-99-Gly-GFP. Transfection was performed 90 minutes before the imaging started. The total imaging time is shown in red in the upper right corner of the movie. After around 6 hours of imaging (i.e. 7 hours and 30 minutes after transfection), a small GFP-positive aggregate is visible in the lower left quadrant. The movie shows imaging data for about 24 hours.

Supplemental Video 2. mutHP-90Gly-GFP-transfected HEK293 cells start to form the first aggregates 14 hours and 30 minutes after transfection. The transfection was performed 4 hours prior to imaging. After around 10 and a half hours of imaging (i.e. 14 hours and 30 minutes after transfection), a small GFP-positive aggregate can be seen just left to the center. The movie shows imaging data from 12 hours.

Supplemental Video 3. The video is an animation of a tomogram generated from CLEM-samples of wtHP-99Gly-GFP-transfected HEK293 cells. The tomogram is showing a 200 nm section with 70 tomographic slices.

Supplementary Figure S1

A Seq_1: mutHP-90Gly-GFP
Seq_2: wtHP-99Gly-GFP

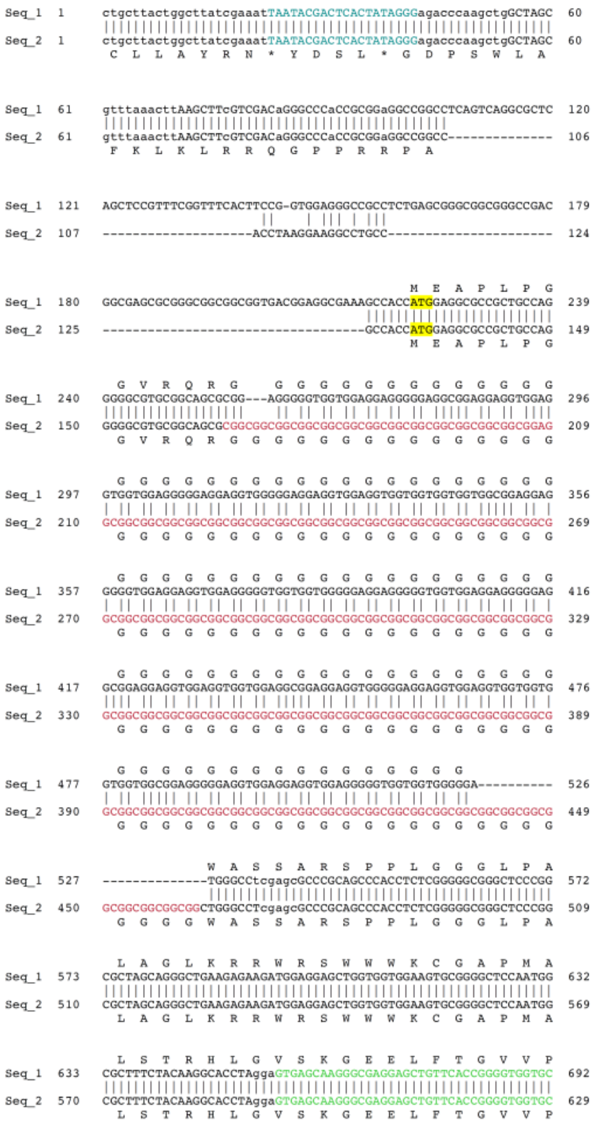

B Seq_1: New mutHP-90Gly-GFP
Seq_2: wtHP-99Gly-GFP

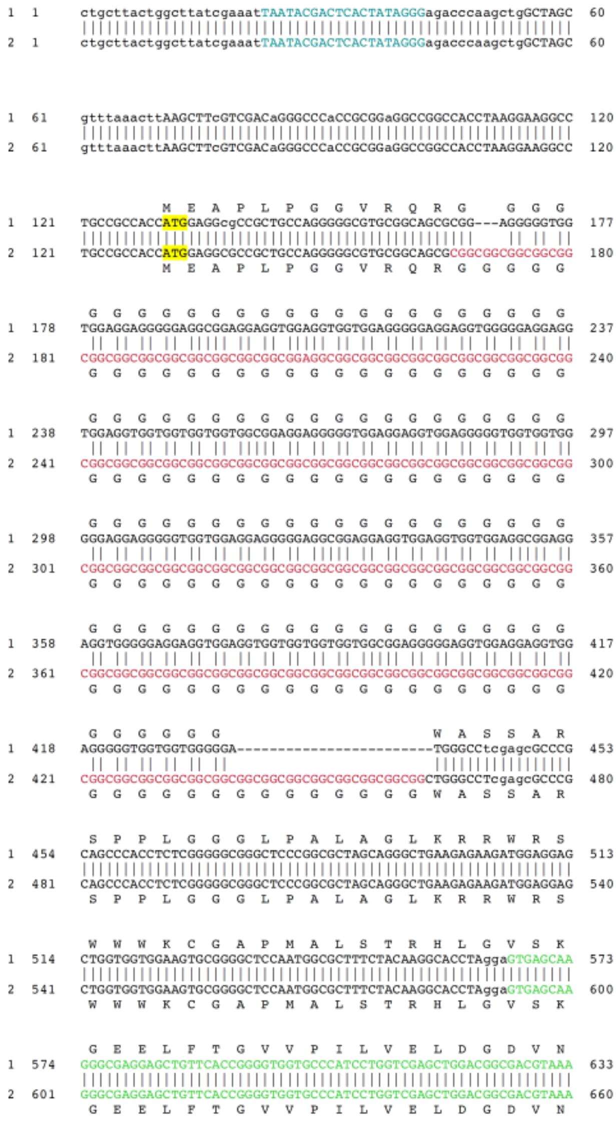

Supplementary Figure S2

A

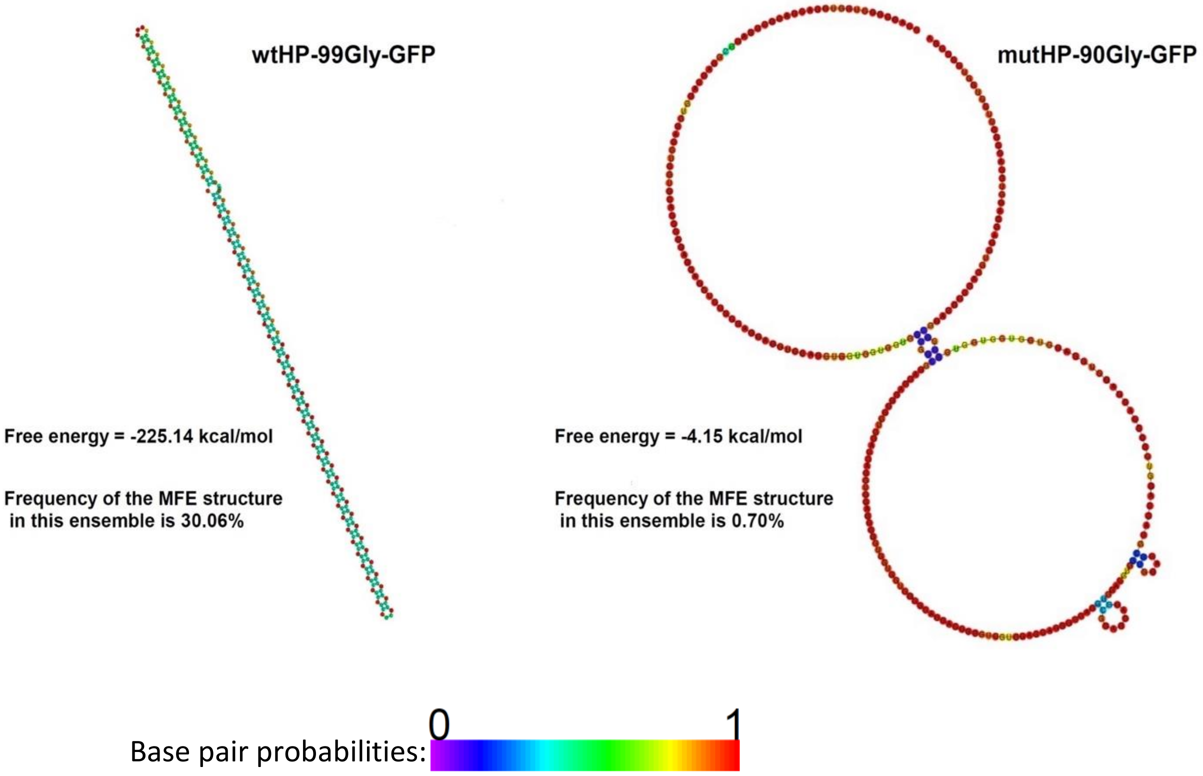

B

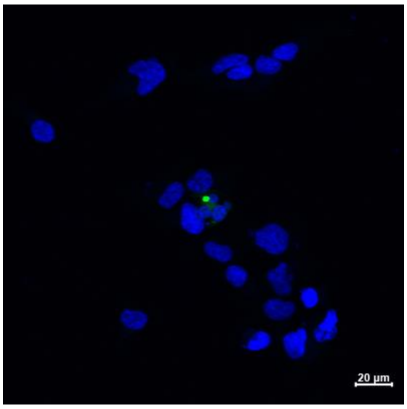

C

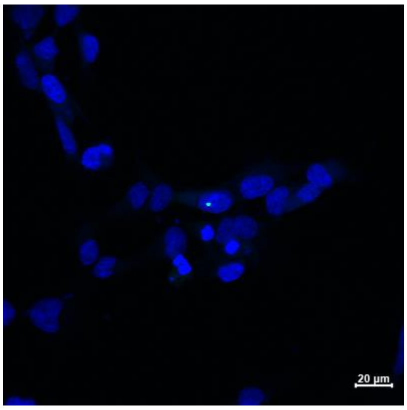

Supplementary Figure S3

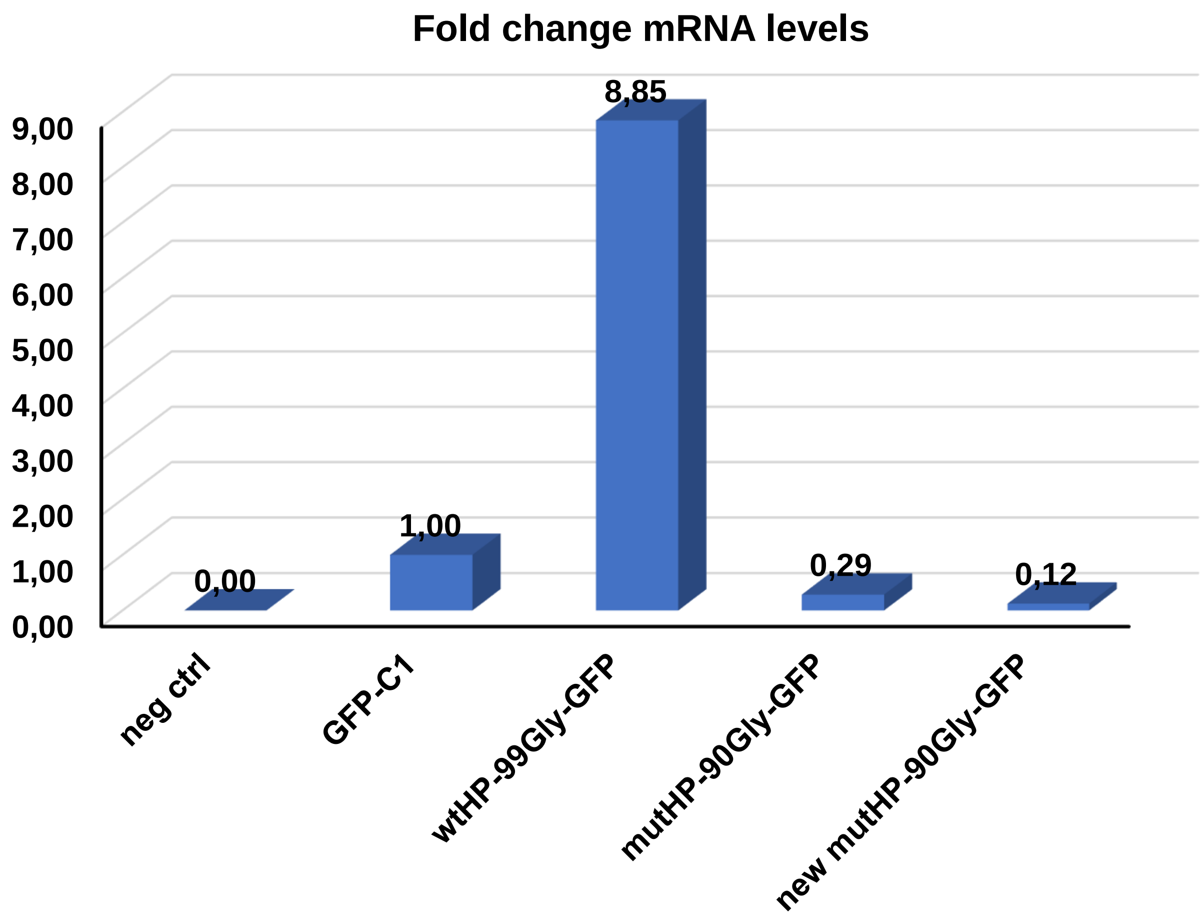

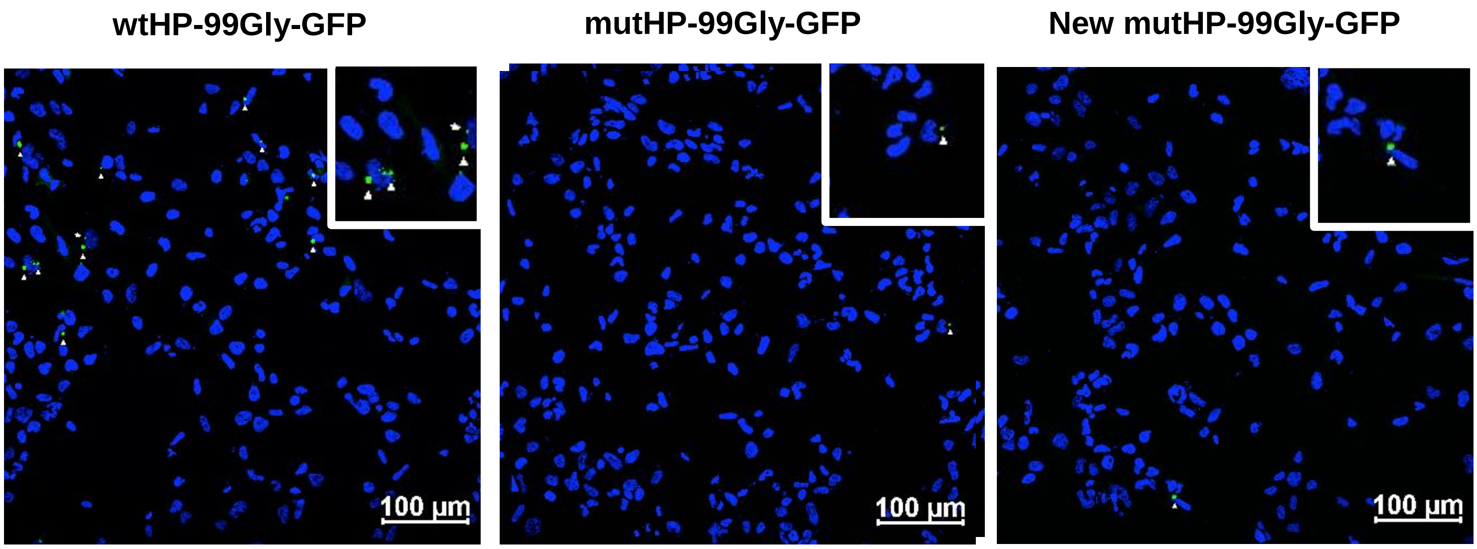

Supplementary Figure S4

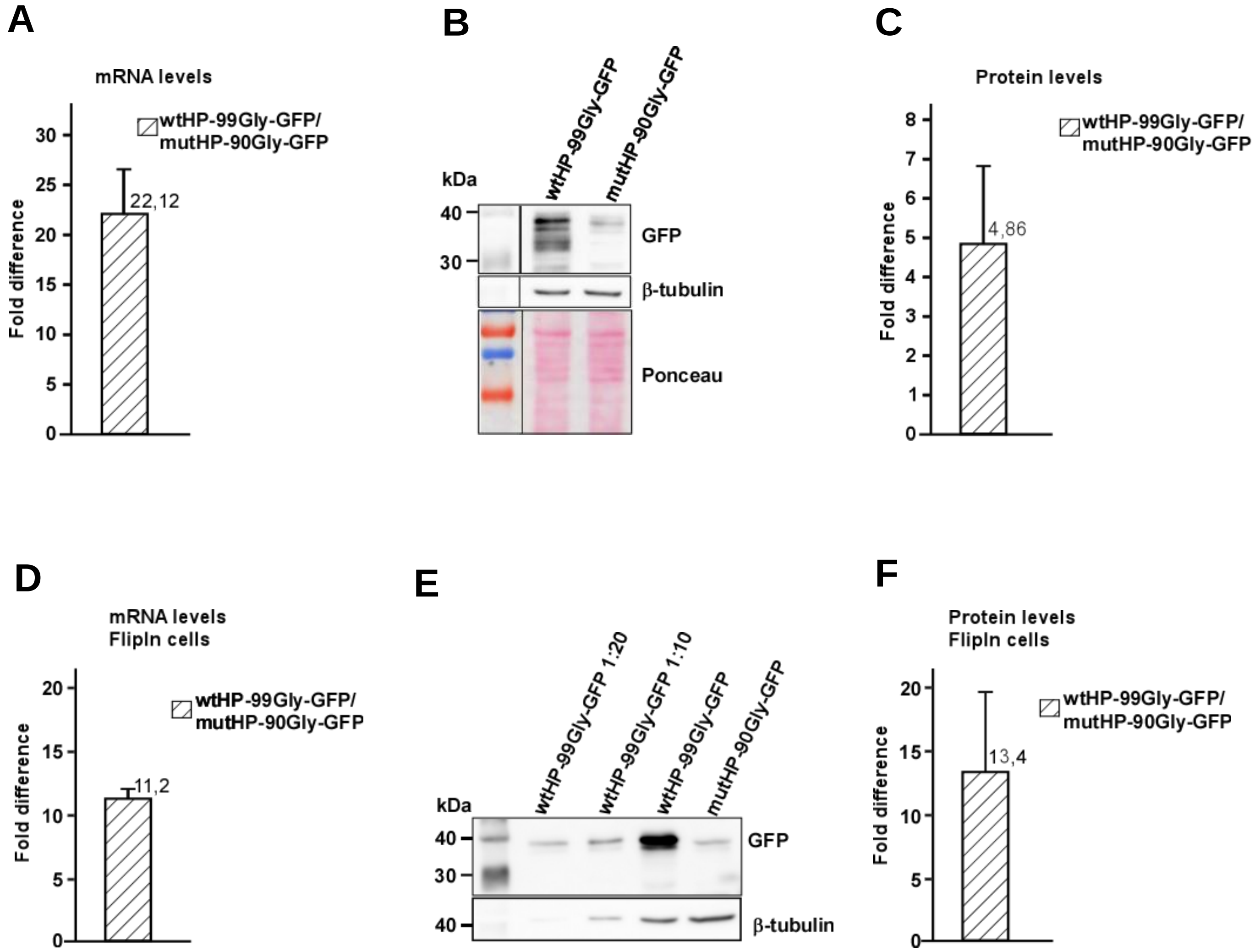

Supplement: Supplementary file 4 [file Data_Sheet_1.pdf]
